# Enhancing local meiotic crossovers in *Arabidopsis* and maize through juxtaposition of heterozygous and homozygous regions

**DOI:** 10.1038/s41477-025-02085-8

**Published:** 2025-09-02

**Authors:** Mikhail E. Mikhailov, Franz Boideau, Maja Szymanska-Lejman, Vasile Botnari, Piotr A. Ziolkowski

**Affiliations:** 1 Laboratory of Plant Resistance, Institute of Genetics, Physiology and Plant Protection, Moldova State University, Chisinau, Moldova; 2 Laboratory of Genome Biology, Institute of Molecular Biology and Biotechnology, Adam Mickiewicz University, Poznan, Poland

**Keywords:** Plant genetics, Plant breeding

## Abstract

Meiotic crossovers, which exchange DNA between homologous chromosomes, are vital for accurate segregation and generate genetic diversity. In plant breeding, they help create new haplotypes by combining beneficial alleles. In *Arabidopsis*, heterozygous regions in an otherwise homozygous background attract more crossovers than in full F_1_ hybrids—a phenomenon so far observed only in this self-fertilizing species. Here we report a similar effect in outcrossing maize: local crossover rates increase up to threefold in regions where polymorphism is spatially confined compared to full hybrids. This stimulation occurs in both male and female meiosis and is strongest when heterozygous regions fully span the measured area, likely due to crossover redistribution. As *Arabidopsis* and maize represent distantly related plant lineages (eudicots and monocots), this shared phenomenon suggests a conserved mechanism. Importantly, it provides a tool for breeding, offering a way to boost recombination and accelerate the introgression of desired traits using interhomologue polymorphism.

## Abstract (web summary)

This study discovered that locally confined DNA differences boost crossover rates in both *Arabidopsis* and maize, revealing a conserved mechanism that can accelerate plant breeding and trait introgression.

## Main

Variation in eukaryotic populations arises through sexual reproduction, which combines genetic information from two parents^1–3^. However, mating is only the final step in creating variation; much of it is established during gamete formation through a specialized cell division process known as meiosis. During meiosis, chromosomes segregate randomly into daughter cells, which contributes to genetic diversity. A key mechanism for further shuffling genetic combinations inherited from parents is the reciprocal exchange of chromosome fragments, known as crossovers^4–6^. Crossovers are essential for crop breeding, and the ability to enhance them locally remains a key challenge in applied genetics^7,8^.

The distribution of crossovers along chromosomes is influenced by a range of factors, many of which remain incompletely understood^9–12^. One such factor is local DNA sequence differences between homologous chromosomes, known as *cis*-acting genetic polymorphism. In most studied species, local polymorphism tends to inhibit crossovers^13–15^. However, in the plant *Arabidopsis thaliana*, crossovers are stimulated in heterozygous regions if these are juxtaposed to homozygous regions on the same chromosome^16–19^. This effect has been most thoroughly studied in *Arabidopsis*, where it was attributed to its compact genome, distinctive recombination landscape and predominantly self-pollinating nature—features that may enable the plant to maximize the genetic benefits of rare outcrossing events^20–24^.

In this Article, through extensive breeding work, we developed a set of nearly isogenic maize lines (NILs) with distinct polymorphism patterns and phenotypic markers, enabling precise measurement of recombination frequency (Rf). Building on a high-quality PacBio genome assembly and high-coverage Illumina sequencing, we discovered that crossover stimulation in heterozygous domains adjacent to homozygous regions occurs even more effectively in maize, showing up to a threefold increase in crossover frequency. Given that maize is naturally outcrossing, with a genome ~20 times larger than *Arabidopsis* and a strikingly different crossover pattering, this finding suggests that the heterozygosity juxtaposition effect may be conserved among flowering plants^25–27^. These results indicate that there may be aspects of plant evolution worth revisiting. Furthermore, as maize is one of the world’s most important crops, our findings offer valuable insights for breeders, presenting a method to direct recombination to specific locations through the simple selection of optimal parental plants. This approach promises a universal strategy for efficiently transferring advantageous traits between crop varieties.

## Results

### Local stimulation of meiotic crossover in polymorphic regions occurs in both *Arabidopsis* and maize

**Fig. 1:**
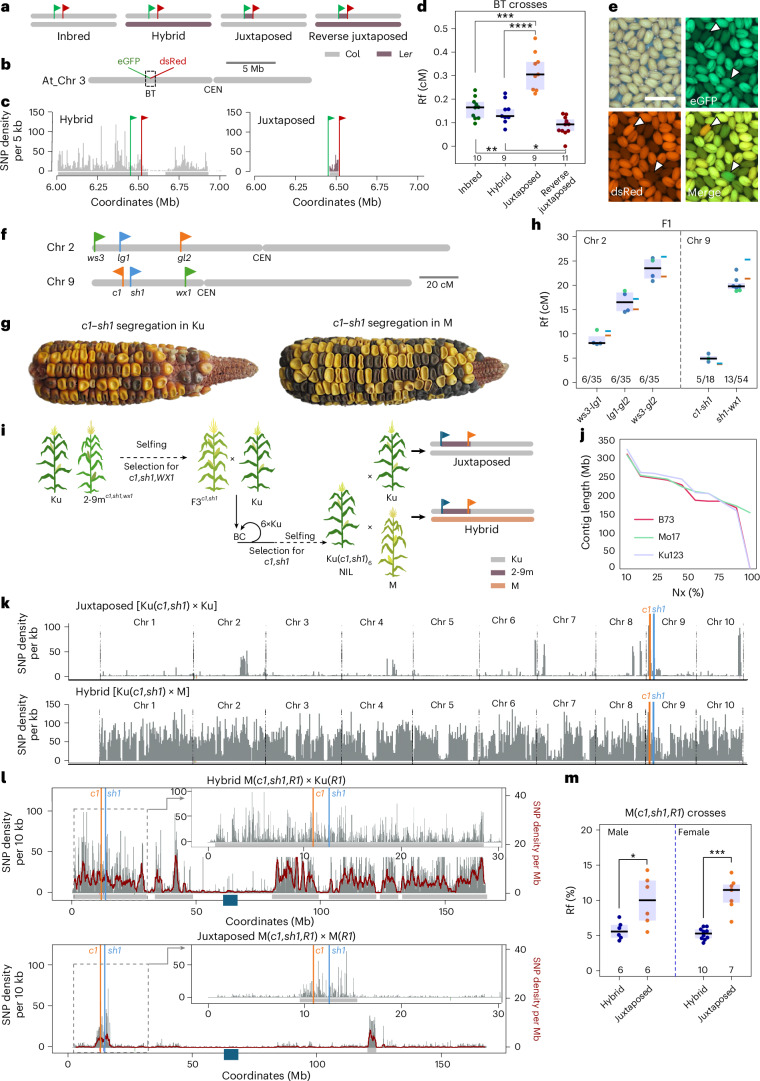
Locally occurring interhomologue polymorphism stimulates crossovers in both *Arabidopsis* and maize. **a**, Interhomologue polymorphism in F_1_ plants representing ‘Inbred’, Hybrid, Juxtaposed and Reverse juxtaposed combinations. Each shows a pair of *A. thaliana* chromosome 3 (At_Chr 3; not to scale), with colour flags representing reporters used to measure crossover rates in the interval. **b**, Ideogram of At_Chr 3, indicating the BT interval location. The dashed rectangle highlights the 1 Mb section shown in **c**. **c**, SNP distribution per 5 kb along a 1 Mb section of At_Chr 3 including the BT interval in the Hybrid and Juxtaposed combinations. Green and red vertical lines show reporter positions. Grey rectangles below the graphs denote heterozygous regions in each configuration. **d**, Rf in the BT interval across the four backgrounds. The *P* values were calculated using Welch’s one-way ANOVA followed by the Games–Howell post hoc test; *P = 0.05, **P = 4.0 × 10^−3^, ***P = 9.9 × 10^−4^, ****P = 4.12 × 10^−4^. Each data point represents a single plant. Box plots: centre line, median; bounds, 25th and 75th percentiles. **e**, Segregation of eGFP and dsRed reporters in F_2_ seeds from Col-BT × L*er*. Arrowheads indicate individual green and red recombinants, corresponding to L*er*/L*er* to Col/L*er* and Col/L*er* to L*er*/L*er* crossover events, respectively. Scale bar, 2 mm. **f**, Marker positions forming the *ws3–lg1–gl2* and *c1–sh1–wx1* intervals on maize chromosomes 2 (Chr 2) and 9 (Chr 9), respectively. Genetic distances (cM) from MaizeGDB. **g**, Maize ears in Ku and M backgrounds showing segregation at *c1* and *sh1* markers. **h**, Rf across five intervals in F_1_ plants (data partly based on Mikhailov and Chernov^31^). Each point is the mean Rf of 4–12 F_1_ families grown in 1 year. Green, M × 2-9m; navy, Ku × 2-9m. Plant numbers indicated below box plots (M/Ku). Box plot centre, median; bounds, 25th and 75th percentiles (see Supplementary Table 5 for details). Expected values from MaizeGDB using Kosambi (blue) and Haldane (orange) models are shown as horizontal bars. **i**, Scheme for generating NILs to measure Rf in maize with different heterozygosity patterns, illustrated with the Ku(*c1*,*sh1*,*R1*) line. *R1* marker, required for *c1* phenotype, not shown. Ku was crossed with 2-9m carrying linked *c1–sh1–wx1* alleles. F_3_ plants homozygous for *c1*, *sh1* and *WX1* were selected and backcrossed seven times to Ku, selecting *c1*,*sh1*/++ heterozygotes. Final plants were selfed and selected for *c1*/*sh1* homozygosity. Crosses to Ku produced Juxtaposed combinations; crosses to M produced fully heterozygous Hybrids. Chromosome 9 configurations are depicted. BC, backcross. **j**, Assembly contiguity is represented as an N_*x*_ plot (the length of the shortest contig that, along with longer and equal-length contigs, represents *x*% of the assembly) for the Ku123 line. **k**, SNP density across the 10 maize chromosomes for Ku(*c1*,*sh1*,*R1*) aligned to pure Ku (top) and M (bottom). Regions of polymorphism reflect introgressions from 2-9m. Orange and blue lines mark *c1* and *sh1* loci. **l**, SNP distribution along chromosome 9 in M(*c1*,*sh1*,*R1*) crosses. Grey bars, SNP density per 10 kb; burgundy line, SNP density per 1 Mb; grey horizontal bars below, estimated heterozygous regions. Coloured lines indicate the *c1–sh1* interval used for Rf. The navy blue square on the *x* axis shows the centromere location. Inset zooms in on interval; SNP density plots partly trimmed. **m**, Rf (%) based on *c1–sh1* marker segregation in male and female backcross generations from Hybrid M(*c1*,*sh1*,*R1*) × Ku(*R1*) and Juxtaposed M(*c1*,*sh1*,*R1*) × M(*R1*) crosses. Each data point denotes one plant. Sample sizes are shown below box plots. The two-sided *P* values were estimated by Welch’s *t*-test; *P = 0.0305, ***P = 4.2 × 10^−4^. Box plot centre, median; bounds, 25th and 75th percentiles.

**Extended Data Fig. 1:**
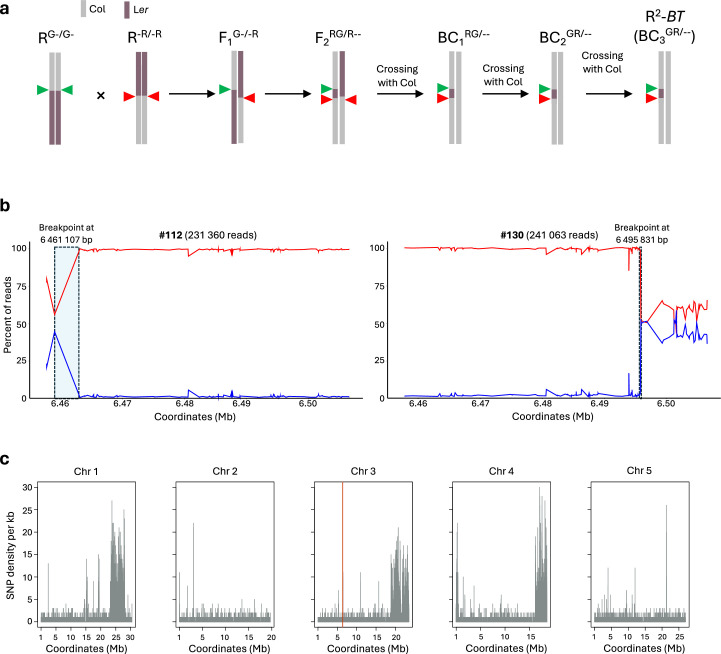
Generation of the R^2^-*BT* line. **a**, Schematic illustrating the creation of R^2^-*BT* through single recombinant crosses between Col-*BT*/L*er* (R) carrying only one reporter gene. Successive backcrosses aim to clean up the L*er* background. **b**, The genetic structure of the *BT* interval for recombinants #112 (R^RG-/G-^, left) and #130 (R^−R/−R^, right) used to create R^2^-*BT*, determined by targeted sequencing of the *BT* region. The plots show the percentage of Col alleles (blue) and L*er* alleles (red) across the entire *BT* region. The dashed rectangles indicate the location of Col/L*er* crossover breakpoints. **c**, The genetic structure of R^2^-*BT* determined through whole-genome sequencing based on short reads. Polymorphisms indicating the presence of L*er*-derived fragments are marked in gray. The *BT* region on chromosome 3 is indicated by a red line.

Segregation of linked markers can be used to measure crossover rates within a defined interval^28–30^. To demonstrate the phenomenon of crossover stimulation in the heterozygous region in *Arabidopsis*, we developed the R^2^-BT line (Recombinant × Recombinant for BT; Extended Data Fig. 1). This was achieved by crossing a recombinant from Col-BT × L*er* cross, which carried a seed-expressed reporter encoding the *Discosoma* red fluorescent protein (dsRed), with another recombinant carrying an enhanced green fluorescent protein (eGFP) reporter, both selected based on previously identified crossover breakpoints^18^. Double-reporter individuals were selected in the F_2_ generation and backcrossed to the Col background, resulting in the R^2^-BT line. This line contained a 35 kb segment between the fluorescent markers derived from the Landsberg *erecta* (L*er*) accession, while the remainder of the chromosome was from Columbia (Col), which is polymorphic to L*er* (Extended Data Fig. 1 and Supplementary Table 1). We then crossed the R^2^-BT line with Col, creating a heterozygous BT interval in an otherwise homozygous background (hereafter ‘Juxtaposed’), and with L*er*, generating a reverse combination, where the BT interval is homozygous and the rest of the chromosome is heterozygous (‘Reverse juxtaposed’) (Fig. 1a,b). In parallel, we generated Col-BT × Col inbred and Col-BT × L*er* hybrid variants as controls (Fig. 1a,b). The crossover frequency in the BT interval was 2.22 times higher in the R^2^-BT × Col Juxtaposed cross compared to the Col-BT × L*er* hybrid (Fig. 1c–e and Supplementary Tables 2 and 3). By contrast, in the R^2^-BT × L*er* Reverse juxtaposed cross, BT crossover frequency was only 0.6 times that observed in the Col-BT × Col inbred (Fig. 1c–e). These results confirm that local heterozygosity within a homozygous background promotes crossovers, whereas local homozygosity within a heterozygous background inhibits them.

To determine whether crossover stimulation in heterozygous regions adjacent to homozygous regions is a general phenomenon in plants, we decided to investigate similar genetic combinations in maize. For this purpose, we used the 2-9m line, which carries a set of linked mutant alleles for phenotypic traits on chromosomes 2 (*ws3*, *lg1* and *gl2*) and 9 (*c1*, *sh1* and *wx1*) (Fig. 1f,g, Supplementary Fig. 1 and Supplementary Table 4). The *c1* marker requires the presence of a dominant *R1* allele on chromosome 10 to show anthocyanin colouration of the kernels. We crossed the 2-9m line with two divergent inbred maize lines: MK01 and Ku123 (hereafter M and Ku, respectively).

**Extended Data Fig. 2:**
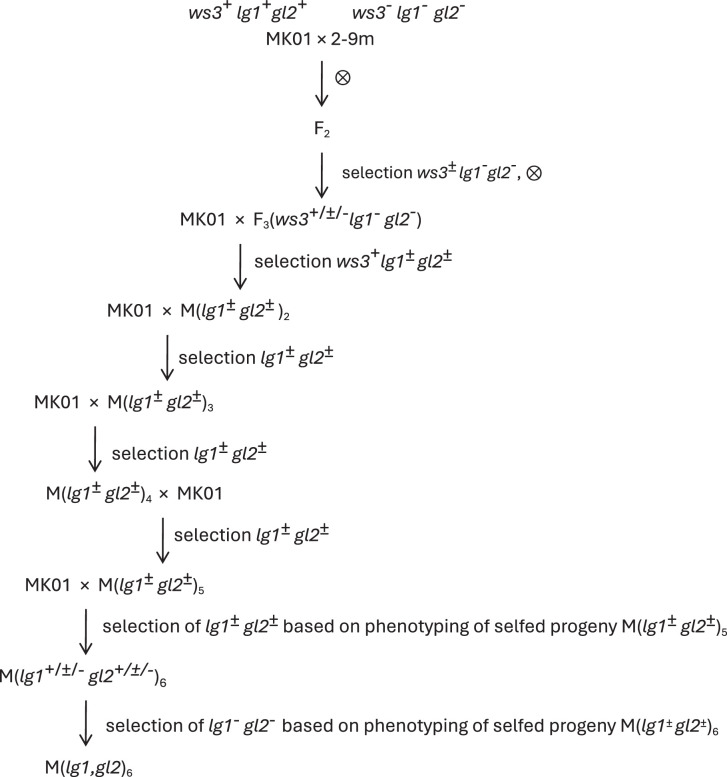
Generation of maize NILs. A detailed scheme illustrating the development of the M(*lg1*,*gl2*)_6_ NIL through successive backcrosses and phenotypic marker selection. ⊗ represents self-pollination. ‘+’ or unlabeled loci indicate homozygosity for the dominant allele, ‘±’ indicates heterozygosity, and ‘-’ indicates homozygosity for the recessive allele. ‘+/±/−’ indicates families with individuals of mixed genotypes. In any MK01 × M(*lg*^±^,*gl*^±^)n families, individuals with genotypes *lg*^+^*gl*^+^, *lg*^+^*gl*^±^, *lg*^±^*gl*^+^, and *lg*^±^*gl*^±^ were identified by analyzing the self-pollinated progeny.

We first examined the Rf in M × 2-9m and Ku × 2-9m F_1_ crosses^31^. For five intervals—*lg1–gl2*, *ws3–lg1* and *ws3–gl2* on chromosome 2, and *c1–sh1* and *sh1–wx1* on chromosome 9—the Rf values were consistent with genetic distances reported by the Maize Genetics and Genomics Database (MaizeGDB)^32^, and no deviations from Mendelian segregation were observed (Fig. 1h and Supplementary Tables 5 and 6). This suggests that the regions of chromosome 2 and 9 covering the examined intervals do not contain extensive chromosomal rearrangements that could affect formation of meiotic crossovers. We then introgressed individual marker combinations into M and Ku through 5–7 backcrosses (Fig. 1i and Extended Data Fig. 2), resulting in the following NILs: M(*c1*,*sh1*,*R1*), Ku(*c1*,*sh1*,*R1*), M(*sh1*,*wx1*), Ku(*sh1*,*wx1*), M(*lg1*,*gl2*), Ku(*lg1*,*gl2*), M(*ws3*,*lg1*), M(*ws3*,*gl2*), M(*c1*,*sh1*,*wx1*,*R1*) and M(*ws3*,*lg1*,*gl2*). In parallel, the M(*R1*) and Ku(*R1*) lines were generated as *R1*-containing variants of the M and Ku lines.

**Extended Data Fig. 3:**
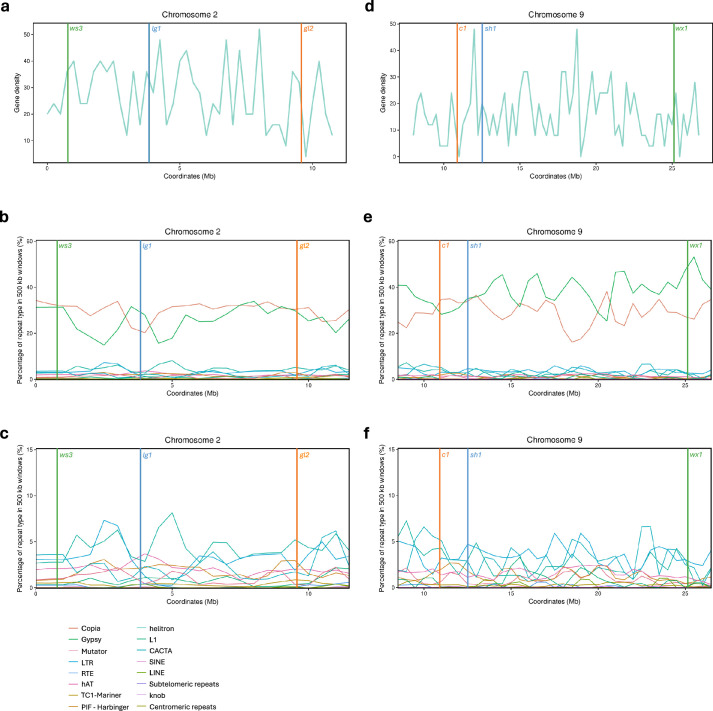
Gene and transposon density in the Ku123 genome regions containing the *ws3-lg1-gl2* and *c1-sh1-wx1* intervals. **a**, Gene density within the *ws3-lg1-gl2* interval, plotted in 250 kb windows. **b**, Transposable element (TE) density within the same interval, plotted in 500 kb windows. **c**, TE density excluding the most common LTRs, *Ty1* (Copia) and *Ty3* (Gypsy). **d-f**, Similar plots as in a-c, but for the *c1-sh1-wx1* interval. Colored vertical lines represent the position of the markers used to measure recombination frequency (Rf).

**Extended Data Fig. 4:**
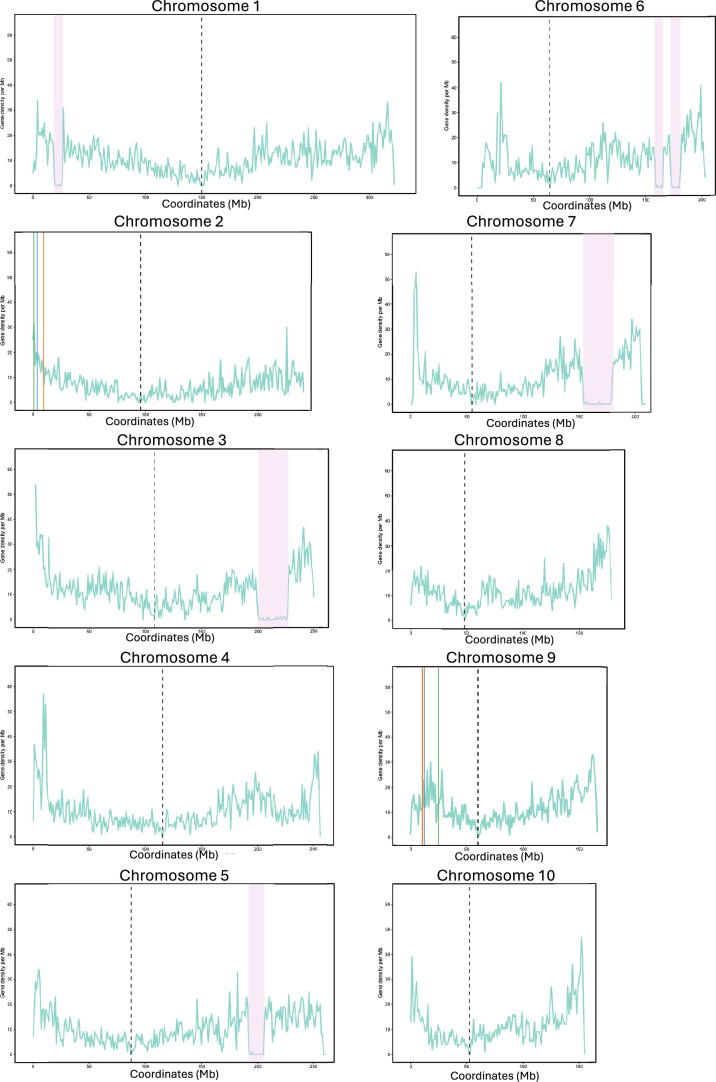
Gene density across the chromosomes of the Ku123 genome assembly. The graphs depict gene density for each chromosome, calculated in 0.5 Mb windows. Light-purple rectangles highlight the positions of heterochromatic knobs. Colored vertical lines represent the position of the markers used to measure Rf.

**Extended Data Fig. 5:**
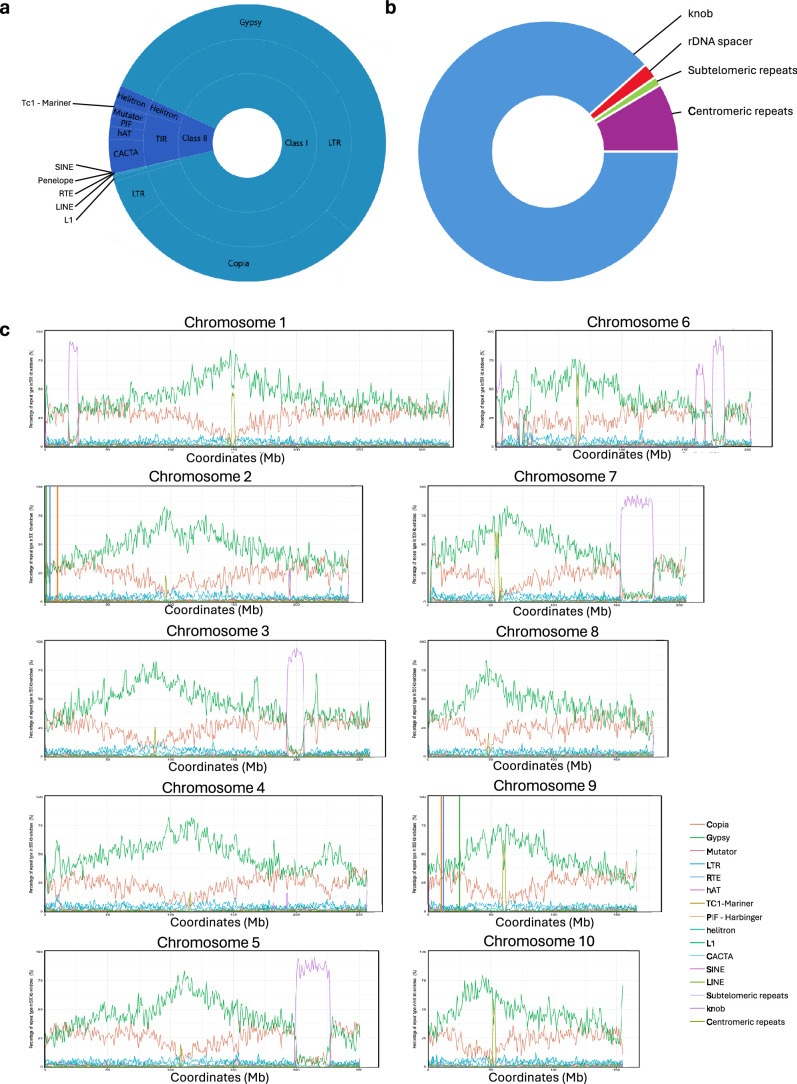
Repeat sequences in the Ku123 genome. **a**, Proportions of different Class I and Class II transposable elements in the Ku123 genome assembly, calculated based on genomic sequence coverage. **b**, Proportions of other repeat types (non-transposable elements) in the Ku123 genome assembly, also calculated based on genomic sequence coverage. **c**, Distribution of various repeat types across the 10 chromosomes of Ku123 genome assembly. Colored vertical lines represent the position of the markers used to measure Rf.

**Extended Data Fig. 6:**
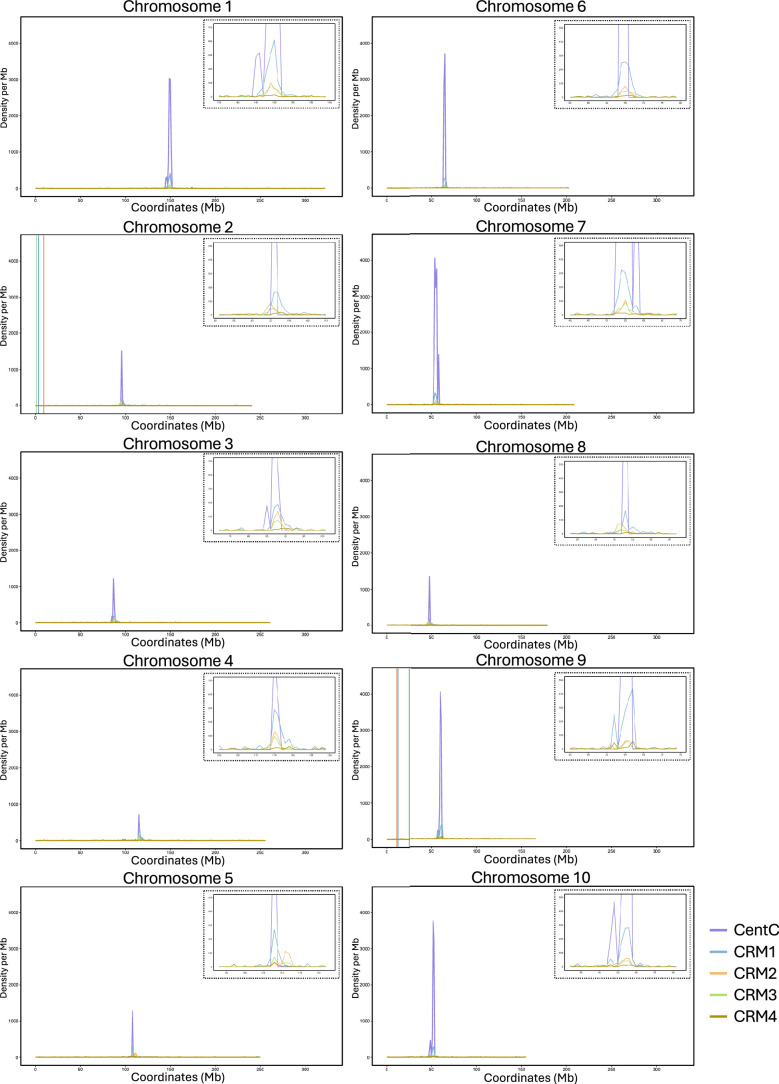
Distribution of the centromeric specific repeats along the chromosomes of the Ku123 genome. For each chromosome, the density per Mb for the centromeric repeats CentC, CRM1, CRM2, CRM3 and CRM4 are represented by the curves colored in light red, light brown, green, blue and purple, respectively. The insets show close-ups of individual centromeric regions. Colored vertical lines represent the position of the markers used to measure Rf.

We used PacBio high-fidelity technology to sequence the Ku parent (at 30× depth) and performed a de novo genome assembly (Fig. 1j and Supplementary Fig. 2). The genome assembly had an N50 value of 241.8 Mb, a total assembly size of 2.432 Gb and a BUSCO (Benchmarking Universal Single-Copy Orthologs) completeness of 98.57% (Fig. 1j, Supplementary Fig. 2 and Supplementary Table 7). Chromosome 9 was represented by two contigs and chromosome 2 by five contigs. In addition, we also identified centromeric regions, genes, repeats and organellar integrants within the Ku assembly (Extended Data Figs. 3–6 and Supplementary Tables 8–11). The region containing the markers *ws3–lg1–gl2* is located at the very end of chromosome 2, at positions 0.78, 3.85 and 9.59 Mb. The *ws3–lg1* interval has a gene density of 24.1 genes per Mb, which is more than twice the genome-wide average of 10.5 genes per Mb, while transposable elements account for 69.1% of this interval compared to the genome average of 81.4%. The *lg1–gl2* interval shows a gene density of 15.5 genes per Mb, with transposable elements comprising 72.9% of the region. The *c1–sh1–wx1* region is also located in a subtelomeric area, although further from the chromosome end, at positions 10.90, 12.52 and 25.14 Mb. The *c1–sh1* and *sh1–wx1* intervals have nearly identical gene densities (16.6 and 16.8 genes per Mb, respectively), but the former contains slightly fewer transposable elements (79.5% versus 82.2%) (Extended Data Figs. 3–5 and Supplementary Table 8).

**Extended Data Fig. 7:**
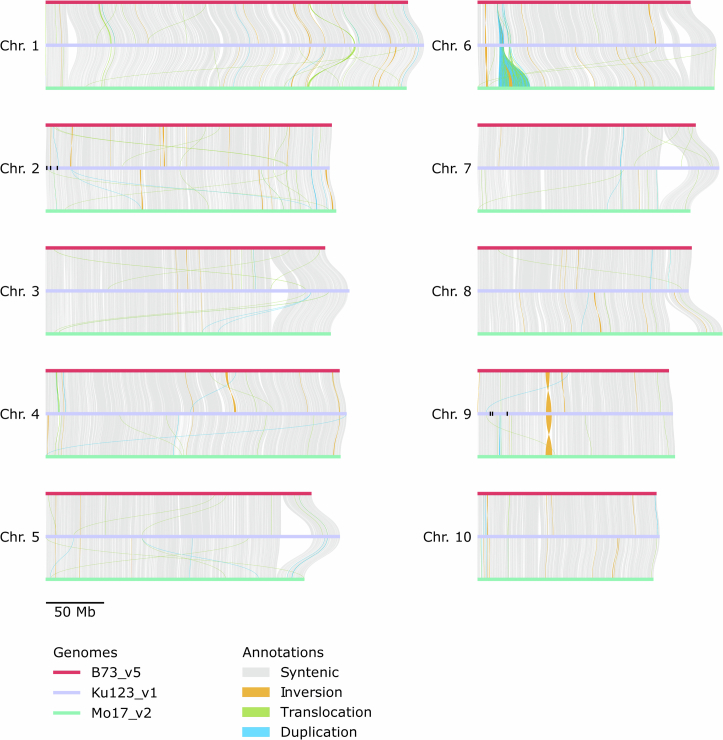
Synteny plots showing the alignment of the Ku123 *de novo* genome assembly with the B73 and M017 reference genomes for every chromosome. Extensive duplication in Mo17 on chromosome 6 corresponds to an expansion of 45S rDNA arrays. Ku123-specific large insertions on chromosomes 1, 3, 5, 6, 7 and specific to M017 on chromosome 8 correspond to knob insertions (see Extended Data Fig. 4). The position of the markers used for recombination frequency (*c1*, *sh1* and *wx1* on chromosome 9 and *ws3*, *lg1* and *gl2* on chromosome 2) are indicated by black tick marks on the Ku123 respective chromosomes.

We then compared the genome assembly of Ku to the reference genome assemblies of the lines B73 and Mo17 (Extended Data Fig. 7)^33,34^. Chromosomes 2 and 9 showed a high collinearity with both reference genomes, with only a few minor structural variations, which should not affect our data. The largest rearrangement detected was a ~4.9 Mb inversion spanning the centromere of chromosome 9. Conversely, Ku has six large heterochromatic knobs with a total length of 84.03 Mb—almost four times the length found in Mo17 (23.47 Mb)^34^. As these knobs are not located directly within the measurement intervals and remain recombinationally inactive, they do not affect our results.

**Extended Data Fig. 8:**
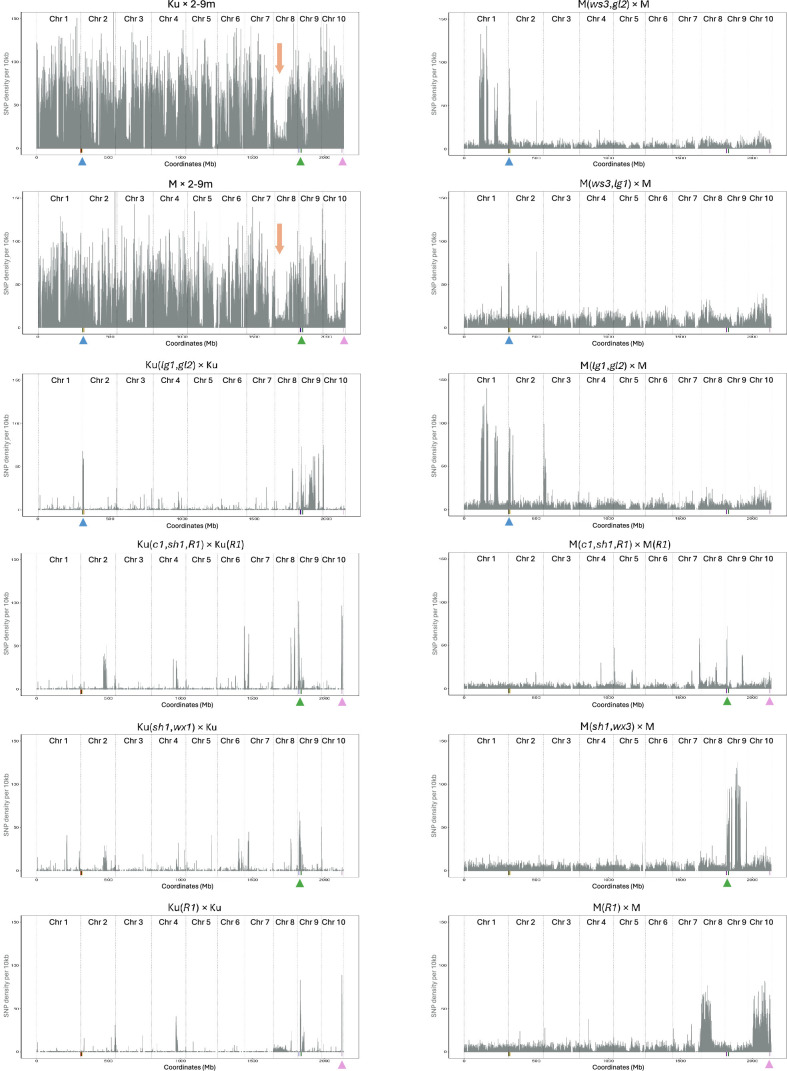
Genome-wide SNP density of the 12 maize lines used in this study. SNP density is represented by gray histograms of 10 kb windows along the 10 chromosomes of each maize line. Arrowheads in blue and green present below the SNP plots indicate the locations of Rf measurement intervals on chromosomes 2 and 9, respectively. The position of the marker *R1* is indicated by a pink arrowhead under the SNP plots on chromosome 10. Orange arrows highlight regions with a reduced density of polymorphisms.

To investigate the effectiveness of introgression within the obtained materials, we sequenced all NILs and their parents using Illumina technology to an average depth of 20× (Supplementary Table 12). The obtained reads were mapped to the Ku assembly, allowing us to examine single-nucleotide polymorphisms (SNP) (Fig. 1k and Extended Data Fig. 8). Chromosome 2 showed a relatively even SNP distribution between the Ku and M lines. By contrast, chromosome 9 contained a large block of approximately 40 Mb with a reduced density of polymorphisms, reflecting a previously described selective sweep^35^. A similar low-density block was also observed when comparing it to the 2-9m line (orange arrows in Extended Data Fig. 8). Importantly, the regions containing the intervals for measuring Rf on both chromosomes were fully polymorphic between M, Ku and 2-9m parents. The analysis confirmed that the NILs carried introgressed regions precisely at the sites encompassing the markers, with additional, unintended introgressions present on other chromosomes (for details, see descriptions of the introgressed regions in the following sections).

Furthermore, each of the NILs was crossed to both the M and Ku parental lines or their *R1*-containing variants. Depending on the parent used, the resulting F_1_ plants were fully heterozygous (for example, Ku(*c1*,*sh1*,*R1*) × M(*R1*)) or heterozygous only for the measurement interval within an otherwise homozygous background (for example, Ku(*c1*,*sh1*,*R1*) × Ku(*R1*)) (Fig. 1i). Hence, these crosses corresponded to heterozygosity/homozygosity combinations equivalent to ‘Hybrid’ or Juxtaposed contexts used in our *Arabidopsis* experiment (Fig. 1a,d). The average SNP densities for the Ku × M and M × 2-9m hybrids were 0.581 and 0.952 SNPs per kb, respectively (Supplementary Table 13). This is substantially lower than in *Arabidopsis* Col × L*er* cross (4.44 SNPs per kb; Supplementary Table 1). As Rf for these F_1_ plants was measured in bidirectional backcrosses—calculated as the ratio of recombinant to total offspring—male and female meiosis was assessed separately.

By analysing the segregation of phenotypic markers in kernels from the progeny of NIL × M and NIL × Ku crosses, we measured the Rf on chromosome 9. We first examined the Rf in the *c1–sh1* interval, located between 16 (*c1*) and 20 (*sh1*) cM of the chromosome 9 genetic map (Fig. 1f). For the M(*c1*,*sh1*,*R1*) line, we observed a nearly twofold increase in Rf, from 5.7 ± 0.5% (*n* = 6, male) and 5.2 ± 0.3% (*n* = 10, female) in Hybrid configuration to 10.0 ± 1.7% (*n* = 6, male) and 10.9 ± 2.1% (*n* = 7, female) in the Juxtaposed configuration (Fig. 1l,m and Supplementary Tables 14 and 15). This result shows that the effect of recombination stimulation in heterozygous regions, when located within an otherwise homozygous chromosome, is not unique to *Arabidopsis* but also occurs in maize, despite differences in polymorphism density.

### The heterozygosity juxtaposition effect occurs in both male and female meiosis and requires adjacent recombinationally active regions

**Fig. 2:**
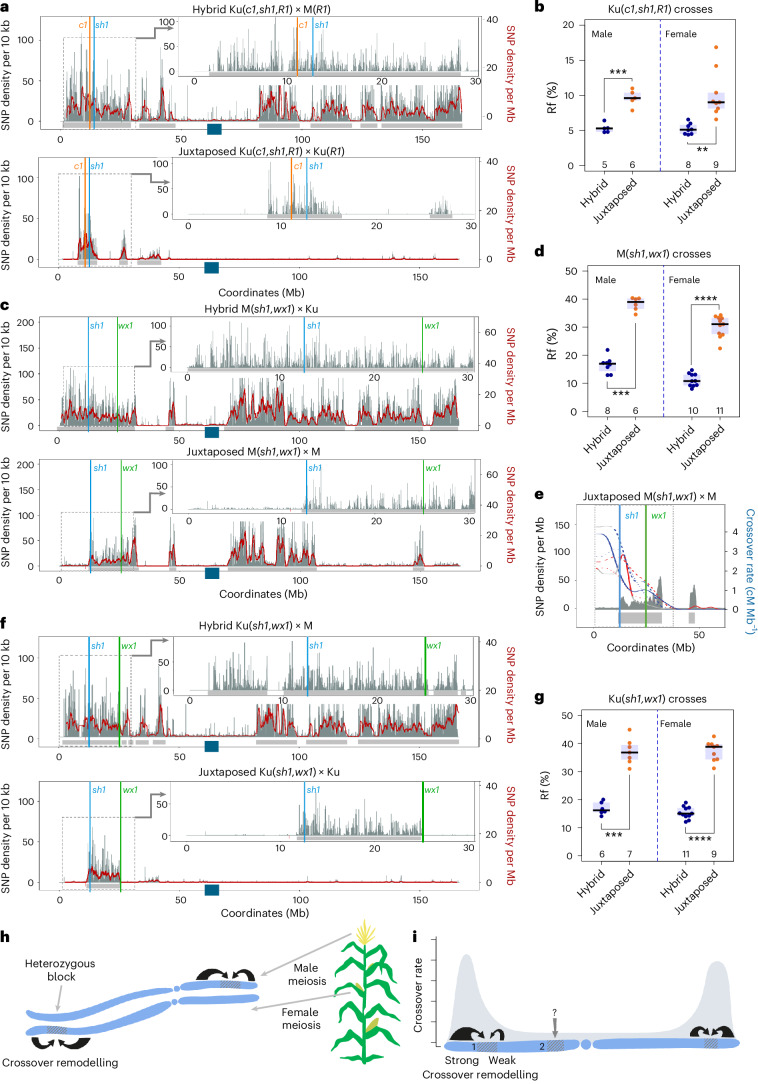
The heterozygosity juxtaposition effect occurs in both male and female meiosis and requires adjacent recombinationally active regions. **a**, Interhomologue SNP distribution in the Ku(*c1*,*sh1*,*R1*) crosses along chromosome 9. Grey vertical bars represent SNP density per 10 kb, and the burgundy trend line shows SNP density per 1 Mb. Grey horizontal bars beneath the graph indicate estimated heterozygous regions based on SNPs. Coloured lines mark the positions of the *c1–sh1* interval used to measure Rf. The navy blue square on the *x* axis shows the centromere location. The inset offers a close-up of the interval, with the SNP density plots below partially trimmed. **b**, Rf (%) measured by segregation of *c1–sh1* markers in male and female backcross generations for Hybrid Ku(*c1*,*sh1*,*R1*) × M(*R1*) and Juxtaposed Ku(*c1*,*sh1*,*R1*) × Ku(*R1*) crosses. The numbers of individuals are indicated below the box plots. Each data point represents measurements from one plant. The two-sided *P* values were estimated by Welch’s *t*-test; ***P = 3.4 × 10^−4^, **P = 2.07 × 10^−3^. The centre line of the box plot shows the median, and the upper and lower bounds show the 75th and 25th percentiles. **c**,**d**, As in **a** and **b**, but for the M(*sh1*,*wx1*) crosses. ***P = 3.6 × 10^−9^, ****P = 9.5 × 10^−11^. **e**, The interhomologue polymorphism to the right of the *sh1–wx1* interval in the Juxtaposed M(*sh1*,*wx1*) × M cross does not affect the Rf within this interval, as it lies within a recombinationally inactive chromosomal region. The grey area shows the distribution of SNPs per Mb, while blue and red lines indicate the crossover frequency (cM Mb^−1^) along the short chromosome arm for Flint × Flint and Dent × Dent crosses, respectively, as reported by ref. ^37^. Solid, dashed and dotted lines represent populations with the highest, median and lowest genome-wide recombination rates among 23 populations within each group^37^. **f**,**g**, As in **a** and **b**, but for the Ku(*sh1*,*wx1*) crosses. ***P = 2.9 × 10^−6^, ****P = 9.2 × 10^−10^. **h**, Model of crossover stimulation in heterozygous regions (shaded) when juxtaposed with homozygous regions (light blue). In maize, this effect is observed in both male and female meiosis. **i**, Polymorphism-triggered crossover stimulation relies on remodelling from recombinationally active chromosomal regions. The increase in crossover frequency in heterozygous region 1 (blue-grey shaded area) is primarily driven by the presence of the upstream homozygous region (large arrow) rather than the downstream homozygous region (smaller arrow), as the former is highly recombinationally active (light-grey shaded area). It remains unknown whether crossovers can be redistributed from recombinationally active regions to inactive regions, such as heterozygous region 2.

To assess whether the effect is specific to the M background, we measured recombination in crosses for the Ku(*c1*,*sh1*,*R1*) line. We observed similar increases, from 5.3 ± 0.3% (*n* = 5, male) and 5.2 ± 0.3% (*n* = 8, female) in the Hybrid configuration to 9.6 ± 1.8% (*n* = 6, male) and 10.1 ± 1.9% (*n* = 10, female) in the Juxtaposed configuration (Fig. 2a,b and Supplementary Tables 14 and 15). Next, we examined the Rf for the *sh1–wx1* interval, located between 20 and 48 cM (Fig. 1f). Here, we observed an even greater increase in Rf in Juxtaposed combinations compared to Hybrid: For the M(*sh1*,*wx1*) lines, the frequency increased from 16.6 ± 1.0% (*n* = 8, male) and 11.2 ± 0.7% (*n* = 10, female) to 38.3 ± 1.0% (*n* = 6, male) and 30.2 ± 1.1% (*n* = 11, female) (Fig. 2c,d and Supplementary Tables 14 and 15). For the Ku(*sh1*,*wx1*) line, the increase was from 16.8 ± 0.9% (*n* = 6, male) and 15.3 ± 0.6% (*n* = 11, female) to 37.2 ± 1.7% (*n* = 7, male) and 37.3 ± 1.2% (*n* = 9, female) (Fig. 2f,g and Supplementary Tables 14 and 15). These results show that, regardless of the genetic background (M or Ku), locally occurring polymorphism between homologues stimulates crossover, leading to a substantial increase in Rf. Importantly, we reveal that the stimulation of crossover due to local interhomologue occurs in both male and female meiosis (Fig. 2h).

As mentioned before, the introgression of *sh1–wx1* to M was much less efficient than into Ku (bottom panels in Fig. 2c,f): Along with the *sh1–wx1* interval, a segment of approximately 6.7 Mb extending to the right from *wx1* was also introgressed in M(*sh1*,*wx1*). In addition, the entire middle part of the chromosome and a fragment of the right arm originated from 2-9m (Fig. 2c). By contrast, in the Ku(*sh1*,*wx1*) line, the introgression is very precise with the end of the region from 2-9m perfectly coinciding with the *wx1* marker (Fig. 2f). Unexpectedly, we observed an equally strong increase in Rf in both cases, with Juxtaposed configurations showing a 2.2- to 2.7-fold increase compared to Hybrid (Fig. 2d,g and Supplementary Tables 14 and 15).

In *Arabidopsis*, the heterozygosity juxtaposition effect depends on the redistribution of crossovers from adjacent homozygous regions into the heterozygous region^16,19^. In maize the crossover frequency, as estimated by chiasma counts, does not differ between hybrids and their parental inbred lines^36^, indicating that the presence of heterozygosity alone does not induce additional crossovers. Therefore, we consider it likely that the juxtaposition effect in maize also results from crossover redistribution. Based on this assumption, one might expect stronger stimulation of recombination in the Ku(*sh1*,*wx1*) line than in M(*sh1*,*wx1*), which, however, is not observed.

**Extended Data Fig. 9:**
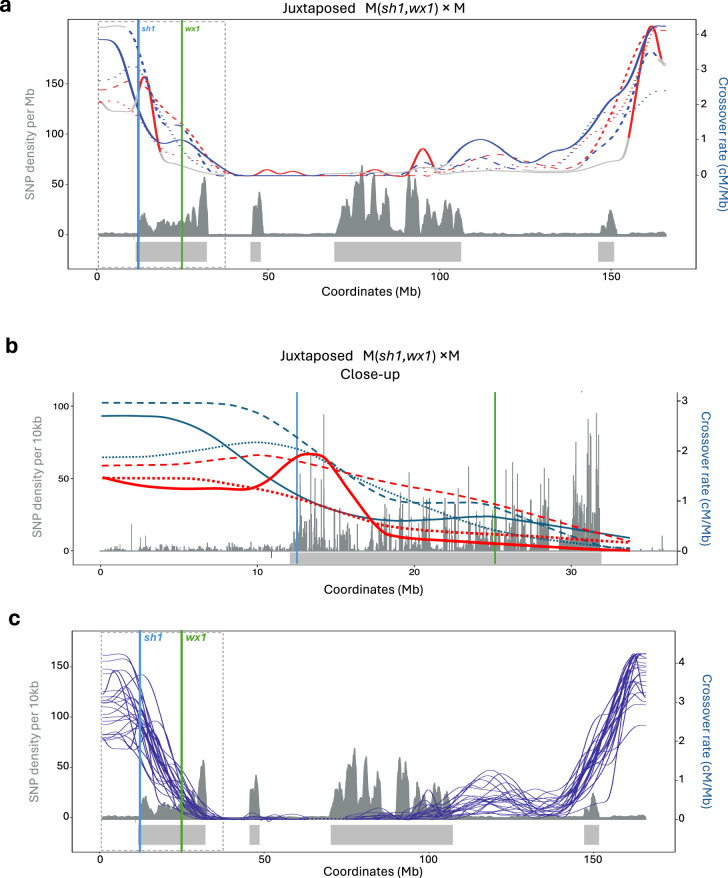
The polymorphism pattern-dependent redistribution of crossovers encompasses only recombinationally active regions. **a**, The interhomolog polymorphism in the ‘Juxtaposed’ M(*sh1,wx1*) × M cross, located to the right of the *sh1-wx1* interval, does not affect the recombination frequency within this interval because it encompasses a recombinationally inactive chromosomal region. The gray area shows the distribution of SNPs per Mb, while blue and red lines indicate the crossover frequency (cM/Mb) along the short chromosome arm for Flint × Flint and Dent × Dent crosses, respectively, as reported by Bauer et al.^37^ Solid, dashed, and dotted lines represent populations with the highest, median, and lowest genome-wide recombination rates among 23 populations within each group^37^. The vertical lines represent the borders of the *sh1-wx1* interval used for measuring Rf. **b**, The distribution of interhomolog SNPs in the M(*sh1,wx1*)_7_ × M cross is shown for the first 35 Mb of chromosome 2. The gray plot shows SNP density per 10 kb and horizontal bars show the estimated distribution of heterozygous regions based on SNPs. The red and blue curves represent the distribution of crossovers as in a. Colored vertical lines indicate the positions of the *sh1-wx1* interval used to measure Rf. **c**, As in a, combining the distribution of crossovers (in cM/Mb, dark blue curves) for 23 different population from Bauer et al.^36^.

We propose that this is because the several-megabase region immediately to the right of *wx1* shows almost no recombination, as evidenced by the marker distribution on the genetic map (Fig. 1f) and the published crossover data^37,38^ (Fig. 2e and Extended Data Fig. 9). We argue that, due to the low Rf in this region, there is limited potential for its redistribution into the heterozygous region. Consequently, the observed increase in the Rf in the juxtaposed M(*sh1*,*wx1*) and Ku(*sh1*,*wx1*) lines is primarily caused by the homozygous regions located to the left side of *sh1–wx1*, which spans nearly 20 cM genetically, despite covering only ~12 Mb (insets in bottom panels in Fig. 2c,f). This result suggests that the juxtaposition effect requires adjacent recombinationally active regions, whereas homozygosity in neighbouring recombinationally silent regions does not contribute to crossover stimulation (Fig. 2i). However, we currently lack the tools to determine whether this mechanism can also stimulate crossovers within inactive regions (Fig. 2i).

### The increase in crossover frequency within the measured interval depends on the distance from the boundary with the homozygous region

**Fig. 3:**
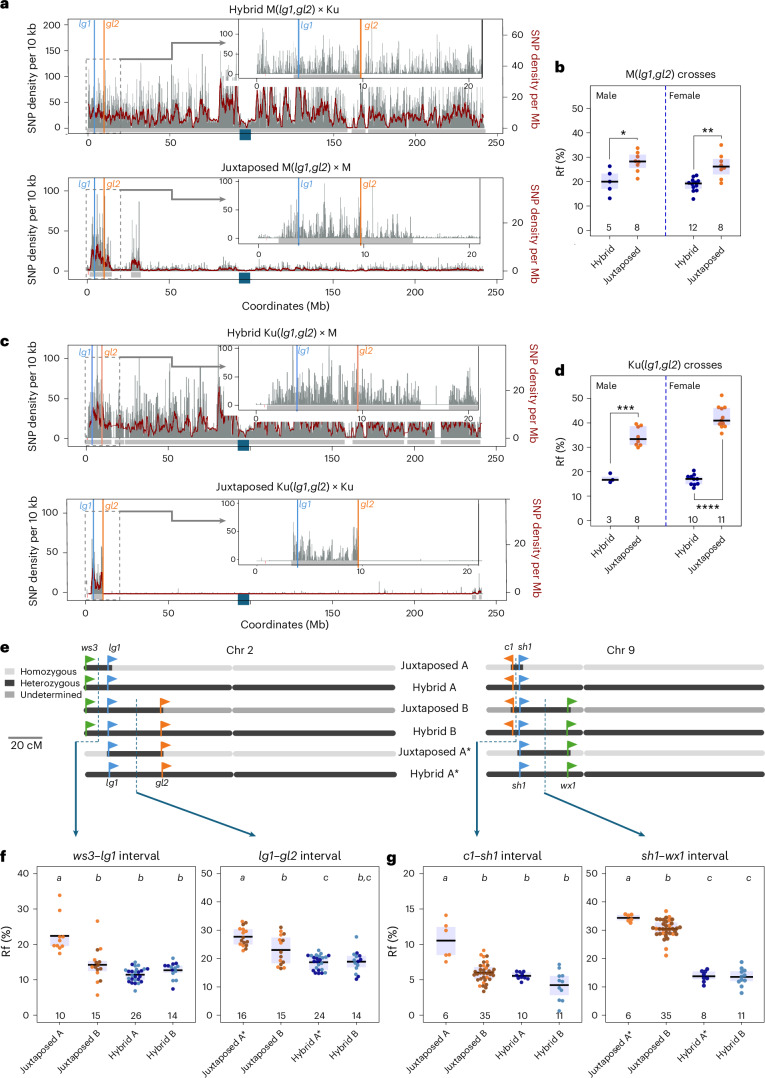
Crossover stimulation in the measurement interval due to the juxtaposition effect is strongest when it closely coincides with the heterozygous region. **a**, Interhomologue SNP distribution in the M(*lg1*,*gl2*) crosses along chromosome 2. Grey vertical bars represent SNP density per 10 kb, and the burgundy trend line shows SNP density per 1 Mb. Grey horizontal bars beneath the graph indicate estimated heterozygous regions based on SNPs. Coloured lines mark the positions of the *lg1–gl2* interval used to measure Rf. The navy blue square on the *x* axis shows the centromere location. The inset offers a close-up of the interval, with the SNP density plots below partially trimmed. **b**, Rf (%) measured by segregation of *lg1–gl2* markers in male and female backcross generations for Hybrid M(*lg1*,*gl2*) × Ku and Juxtaposed M(*lg1*,*gl2*) × M crosses. The two-sided *P* values were estimated by Welch’s *t*-test; *P = 0.0196, **P = 0.0029. **c**,**d**, As in **a** and **b**, but for the Ku(*lg1*,*gl2*) crosses. ***P = 1.2 × 10^−5^, ****P = 9.2 × 10^−11^. **e**, Genetic structure of the combinations used in crosses for the intervals *ws3–lg1–gl2* (chromosome 2) and *c1–sh1–wx1* (chromosome 9). For chromosome 2, Juxtaposed A corresponds to the cross M × M(*ws3*,*lg1*), Juxtaposed B to M × M(*ws3*,*lg1*,*gl2*), and Juxtaposed A* to M × M(*lg1*, *gl2*). For the Hybrid category, combinations A, B and A* are the same as for the Juxtaposed category but crossed with Ku. For chromosome 9, Juxtaposed A, B and A* correspond to M × M(*c1*,*sh1*,*R1*), M × M(*c1*,*sh1*,*wx1*,*R1*) and M × M(*sh1*,*wx1*), respectively, while the Hybrid combinations are analogous but involve crosses with Ku. The diagram indicates only the approximate positions of heterozygous regions. Detailed polymorphism patterns for the Juxtaposed A and A* configurations are shown in **a**, and in Figs. 1l and 2c. For the Juxtaposed B combinations, precise sequence data are not available; however, the homozygous regions are expected to span large portions of the chromosomes outside the measurement intervals, as the lines were generated through six to eight rounds of backcrossing. **f**, Rf in the *ws3–lg1* and *lg1–gl2* intervals for the combinations shown in **e**. Statistical significance was determined using two-sided Welch’s ANOVA, followed by the Games–Howell post hoc test (see Supplementary Table 17 for details). **g**, Similar to **f**, but depicting the *c1–sh1* and *sh1–wx1* intervals for the genotype combinations presented in **e**. For **b**, **d**, **f** and **g**, the number of individuals is indicated below the box plots. Each data point represents measurements from a single plant. Different colours within the same group indicate data collected in different years. The centre line of each box plot represents the median, and the upper and lower bounds correspond to the 75th and 25th percentiles, respectively.

Based on the segregation of phenotypic markers observed in seedlings, we measured the Rf in intervals located on chromosome 2 (Fig. 1f). In the *lg1–gl2* interval, we again observed an increase in the Rf in Juxtaposed compared to Hybrid configurations (Fig. 3a–d). For M(*lg1*,*gl2*) line, this increase was from 20.0 ± 2.3% (*n* = 5, male) and 18.8 ± 0.8% (*n* = 12, female) to 28.1 ± 1.4% (*n* = 8, male) and 26.4 ± 1.8% (*n* = 8, female), respectively. However, this increase was much more pronounced in the Ku(*lg1*,*gl2*) line, rising from 17.3 ± 1.1% (*n* = 3, male) and 16.7 ± 0.7% (*n* = 10, female) to as high as 34.5 ± 1.4% (*n* = 8, male) and 42.3 ± 1.4% (*n* = 11, female), respectively (Fig. 3a–d and Supplementary Tables 14 and 15).

To understand this difference, we examined the heterozygosity pattern in M(*lg1*,*gl2*) and Ku(*lg1*,*gl2*). We observed that while the introgression in Ku almost perfectly coincides with the interval, the introgression in M extends approximately 7 Mb beyond the *lg1–gl2* region (bottom panels in Fig. 3a,c). Consequently, the crossover redistribution from homozygous to heterozygous regions also encompasses the area outside the measurement interval, leading to a lower increase in recombination rates within this interval in the M background compared to the Ku background. Thus, our results show that the stimulation of crossover recombination is more pronounced when the measurement region closely coincides with the heterozygous region.

To further investigate this relationship, we used a series of three-point crosses in which Rf was measured in two adjacent intervals on the same chromosome and compared the results with data from two-point crosses (Fig. 3e). Based on the markers, we classified the crosses into three Juxtaposed types—A, A* and B—where A and A* have the shortest and B the longest heterozygous region. Similarly, hybrid crosses were categorized into types A, A* and B, although they remained fully heterozygous and differed only in the parental origin of the region containing the markers (from genotype M or 2-9m). On this occasion, we also compared the kernel set between the Juxtaposed and Hybrid combinations and found no differences indicative of reduced fertility (Supplementary Table 16). Rf was measured for all marker combinations (see Methods for details).

In all cases, ‘Juxtaposed A’ or ‘Juxtaposed A*’ combinations showed significantly higher Rf than ‘Juxtaposed B’ and both Hybrid types (Fig. 3f,g and Supplementary Tables 14 and 17). Moreover, for the longer intervals (*lg1–gl2* and *sh1–wx1*), Juxtaposed B combinations also showed significantly higher Rf than at least one of the Hybrid types, indicating that extending the heterozygous region beyond the measurement interval reduces the magnitude of crossover stimulation (Fig. 3f,g and Supplementary Table 17). By contrast, for the shorter intervals *ws3–lg1* and *c1–sh1*, where the heterozygous region is several times longer than the measurement interval, the Juxtaposed B combinations showed recombination frequencies that did not differ significantly from those observed in the hybrids (Supplementary Table 17). These findings suggest that crossover stimulation is stronger when the measurement interval is closer to the boundary with a homozygous region. However, an alternative hypothesis remains plausible: that the juxtaposition effect increases as the length of the heterozygous block decreases.

**Fig. 4:**
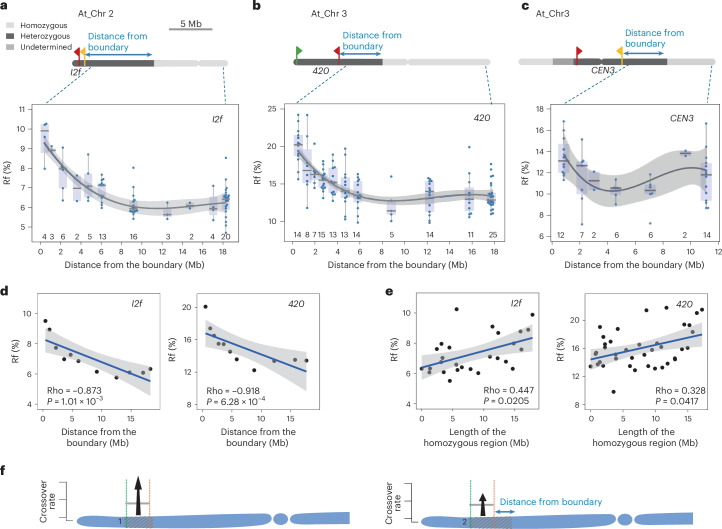
Local crossover frequency increases with proximity to the boundary with the homozygous region. **a**–**c**, Crossover frequency in F_2_ individuals from Col-0 × Ct-1 populations for the *I2f* (**a**), *420* (**b**) and *CEN3* (**c**) intervals, plotted as a function of the distance between the heterozygous measurement interval and adjacent homozygous regions. The top panels show *Arabidopsis* chromosomes with the positions of all three intervals and illustrate how the distance from the boundary is calculated. Chromosomes 2 and 3 in **a** and **c** were inverted to match the orientation of the data shown in the bottom panels. The bottom panels show Rf (%) for plants grouped by distance between the interval and the boundary. For *CEN3* (**c**), only the effect on the long chromosome arm was analysed. Each data point represents an individual F_2_ plant. The centre line of each box plot indicates the median, and the box edges represent the 25th and 75th percentiles. Whiskers indicate the data range within 1.5× the interquartile range from the lower and upper quartiles. The trend line represents a third-degree polynomial fit generated using linear regression. Grey area shows 95% confidence interval of the fitted polynomial model. **d**, Spearman rank correlation between Rf (cM) and the distance of the heterozygous measurement interval (*I2f* or *420*) from the boundary with the adjacent homozygous region. Each data point represents the mean Rf for a group of samples sharing the same distance from the boundary. A linear regression model was applied to illustrate the overall trend, with the grey area showing the 95% confidence interval. **e**, As in **d**, but for the correlation between Rf and the length of the adjacent homozygous region. **f**, The influence of the genetic structure of the studied region on Rf. Heterozygous regions are shown as light-grey shaded areas in otherwise homozygous chromosome. The intervals used for recombination measurements are indicated by coloured dashed lines, and the average Rf in these intervals for full hybrids is represented by the grey bars. When the studied interval overlaps with the heterozygous region (situation 1), the increase in the interval crossover frequency will be higher than when the heterozygous region extends beyond the studied area (situation 2).

To differentiate between these possibilities, we reanalysed data from three *A. thaliana* Col-0 × Ct-1 F_2_ populations for which more detailed information was available^16^. In these datasets, each F_2_ individual was genotyped along the chromosome, allowing us to more precisely define the length and position of the heterozygous region encompassing the measurement interval. Crossover frequency was quantified for each individual based on the segregation of fluorescent markers in pollen (*I2f* and *CEN3* populations) or seeds (*420* population) (Supplementary Table 18). We then grouped individuals according to the distance between the measurement interval and the boundary with the adjacent homozygous region and plotted crossover frequencies for each F_2_ plant within these groups (Fig. 4a–c). For the *I2f* and *420* populations, where the measurement intervals are located at the ends of chromosomes, we performed Spearman correlation analysis using group-wise average recombination frequencies (Fig. 4d). We found that the closer the interval was to the homozygous region, the stronger the crossover stimulation effect (Spearman *Rho* = −0.92, *P* = 6.29 × 10^−4^, and −0.87, *P* = 1.01 × 10^−3^, for *420* and *I2f* intervals, respectively).

Next, we regrouped individuals based on the length of the homozygous block directly adjacent to the heterozygous region containing the measurement interval. While we observed that longer homozygous segments were associated with stronger recombination stimulation within the neighbouring heterozygous region, the correlations were notably weaker (Rho = −0.45, *P* = 0.02 for *420*; Rho = −0.33, *P* = 0.042 for *I2f*; Fig. 4e). Based on these results, we conclude that the key determinant of the juxtaposition effect is the physical proximity of the heterozygous region to the homozygous boundary (Fig. 4f). These findings also indicate that even a relatively short homozygous segment adjacent to a heterozygous region can substantially enhance recombination in the latter.

For the *CEN3* population, correlation analysis was not feasible because the measurement interval is located in the middle of the submetacentric chromosome, meaning that the heterozygous region can extend toward both chromosome arms (Fig. 4c). However, when plotting crossover frequency as a function of distance to the boundary for a subset of individuals with a partially or fully fixed short arm, we observed that the juxtaposition effect also operates within centromeric regions. It is important to note that the data presented here are from *A. thaliana*, and it remains an open question whether heterozygosity-juxtaposition-triggered crossover stimulation also occurs in pericentromeric regions in maize.

### Short homozygous blocks interrupting the heterozygous region suppress local crossover stimulation

**Fig. 5:**
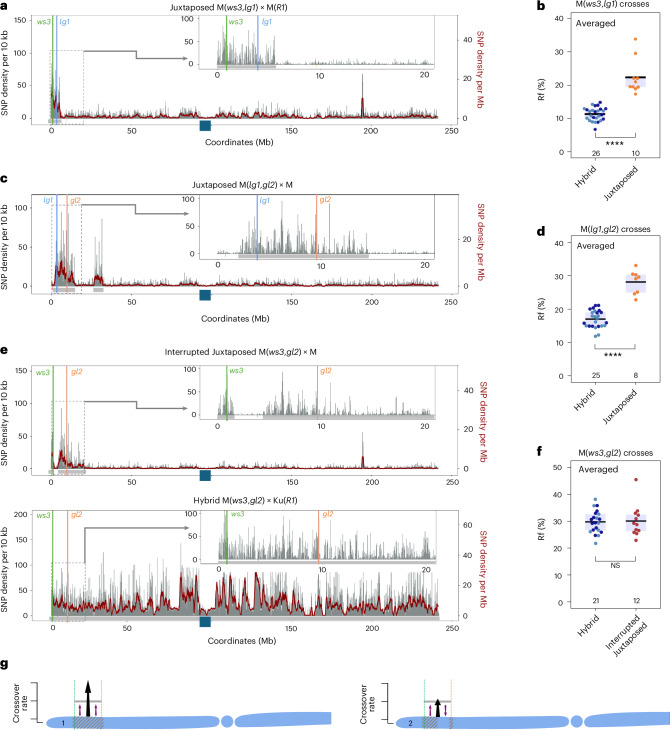
Short homozygous blocks interrupting the heterozygous region suppress local crossover stimulation. **a**, Interhomologue SNP distribution in the M(*ws3*,*lg1*) × M cross along chromosome 2. Grey vertical bars represent SNP density per 10 kb, with the burgundy trend line showing SNP density per 1 Mb. Grey horizontal bars beneath the graph indicate estimated heterozygous regions based on SNPs. Coloured lines mark the positions of the *ws3–lg1* interval used to measure Rf. The navy blue square on the *x* axis shows the centromere location. The inset offers a close-up of the interval, with the SNP density plots below partially trimmed. **b**, Rf (%) measured by segregation of *ws3–lg1* markers in F_2_ generations for Hybrid M(*ws3*,*lg1*) × Ku(*R1*) and Juxtaposed M(*ws3*,*lg1*) × M crosses (male/female averaged). The two-sided *P* values were estimated by Welch’s *t*-test; ****P = 7.1 × 10^−5^. The numbers of individuals are indicated below the box plots. Each data point represents measurements from one plant. Different colours within the same group indicate data collected in different years. The centre line of the box plot shows the median, and the upper and lower bounds show the 75th and 25th percentiles. **c**,**d**, As in **a** and **b**, but for the M(*lg1*,*gl2*) crosses. ****P = 1.3 × 10^−5^. **e**, As in **a**, but for the Hybrid M(*ws3*,*gl2*) × Ku(*R1*) and Interrupted juxtaposed M(*ws3*,*gl2*) × M crosses, where the heterozygous region contains a short homozygous block (gap). **f**, As in **b**, but measured by segregation of *ws3–gl2* markers. NS, not significant. **g**, The effect of genetic structure on Rf. Heterozygous regions are shown as light-grey shaded areas in otherwise homozygous chromosome. The intervals used for recombination measurements are indicated by coloured dashed lines, and the average Rf in these intervals for full hybrids is represented by the grey bars. Small violet arrows denote expected subsegmental crossover changes based on homozygous/heterozygous state. Crossover frequency is lower when heterozygosity is interrupted by homozygosity (compare chromosome configurations 1 and 2).

Finally, we examined recombination in maize by investigating segregation of *ws3*, *lg1* and *gl2* markers in the F_2_ generation (Figs. 1f and 5a–f). For the M(*ws3*,*lg1*) and M(*lg1*,*gl2*) introgressions, we observed a statistically significant increase in Rf in the Juxtaposed combination (cross with M) compared to Hybrid combination (cross with Ku) (*P* = 7.1 × 10^−5^ and *P* = 1.3 × 10^−5^, respectively, Welch’s test; Fig. 5a–d and Supplementary Tables 14 and 15). However, when we introduced a homozygous block of approximately 3 Mb within the heterozygous *ws3–gl2* interval, forming the ‘Interrupted juxtaposed’ combination (top panel in Fig. 5e), the Rf did not differ from that observed in the full hybrid (Fig. 5e,f).

As both the *ws3–lg1* and *lg1–gl2* intervals show increased recombination when juxtaposed with homozygous regions (Fig. 5b,d), the *ws3–gl2* interval would also be expected to show elevated recombination. The absence of this effect in the Interrupted juxtaposed configuration likely reflects the mechanism observed in *Arabidopsis*, where the juxtaposition effect relies on crossover redistribution from homozygous to neighbouring heterozygous regions (see also the comparison between Juxtaposed with Reverse juxtaposed in Fig. 1d). In the Interrupted juxtaposed configuration, crossover events may be redirected from the intervening homozygous block toward neighbouring heterozygous regions within the same measuring interval, ultimately yielding no significant change in the *ws3–gl2* Rf (Supplementary Table 15). This outcome is therefore consistent with findings in *Arabidopsis*^16^.

## Discussion

In yeast and mammals, local interhomologue polymorphism inhibits crossover recombination, leading to repair via alternative mechanisms^13,39–41^. By contrast, crossover stimulation in heterozygous regions adjacent to homozygous chromosome segments has been well documented in *Arabidopsis*^16–19^. It is important to note that *A. thaliana* predominantly reproduces through self-pollination, resulting in a high frequency of homozygous loci in natural populations^42,43^. Therefore, this stimulation of crossover in heterozygous regions is often interpreted as a strategy to maximize the use of remaining genetic variation and enhance population variability^44,45^. In addition, *Arabidopsis* has several unique features, including short life cycle, a small, compact genome, low densities of DNA methylation and transposable elements, a structurally conserved genome and a high correlation between SNPs and recombination as well as a crossover distribution concentrated toward chromosome centres^17,20,46^. These characteristics sharply contrast with those of larger crop genomes, such as wheat, barley and maize^26,47–49^. Surprisingly, in this study, we provide direct evidence that this crossover stimulation effect also occurs in maize, which is naturally outcrossing.

Maize has long served as an excellent model for studying meiotic recombination, owing to its remarkable phenotypic variability and the pioneering work of many researchers, most notably Barbara McClintock. For over a century, research using maize has revealed that most crossovers occur within genes, while the abundant retrotransposons present in its genome are largely recombinationally inactive^50^. Due to the high variability of retrotransposons and their dominant contribution to the physical length of chromosomal regions, much of the research has focused on their suppressive effect on recombination^51,52^. For instance, heterozygosity for a 26 kb retrotransposon cluster insertion leads to a twofold reduction in Rf between the *bz1* and *stc1* markers^53^.

Importantly, intragenic recombination studies have enabled the separation of the effects of repetitive elements from those of SNP^54,55^. The most extensive investigation was carried out by Dooner within the highly recombinogenic *Bronze* (*Bz*) hotspot, which spans approximately 1.5 kb (ref. ^55^). By using flanking markers on both sides of the *Bz* locus, the experimental system enabled the detection of both crossover and non-crossover recombination events (that is, short, non-reciprocal exchanges). Comparing different crosses involving *bz* alleles with varying degrees of polymorphism, Dooner observed that crossovers predominated in polymorphic allele pairs, whereas both crossover and non-crossover events were observed when sequence polymorphism was minimal. This suggests that polymorphism within a single hotspot may bias repair toward the crossover pathway—an observation consistent with recent findings in *Arabidopsis*^18^. Unfortunately, the available data do not allow a direct comparison of crossover frequency between the different sets of alleles.

An early indication that heterozygosity flanked by homozygous regions might stimulate crossover frequency in maize came from the study by Mikhailov and Chernov^31^. They examined F_2_ populations and measured Rf between pairs of markers, with the extent of heterozygosity inferred using a third marker. Recombination was higher in crosses where heterozygosity was confined to the intervals defined by the two markers. These results served as a foundation for the current study.

Comparable phenomena have not been documented in other plant species, with the possible exception of lima bean (*Phaseolus lunatus*), as reported by Allard^56^. In that study, five generations of inbreeding were carried out while maintaining heterozygosity in three marker-defined intervals. Although the author aimed to select for plants with altered recombination frequencies in both positive and negative directions, only selection for increased recombination was successful, while attempts to decrease Rf were ineffective. This outcome aligns with the juxtaposition effect, as inbreeding would have progressively increased homozygosity in genomic regions outside the monitored intervals.

**Fig. 6:**
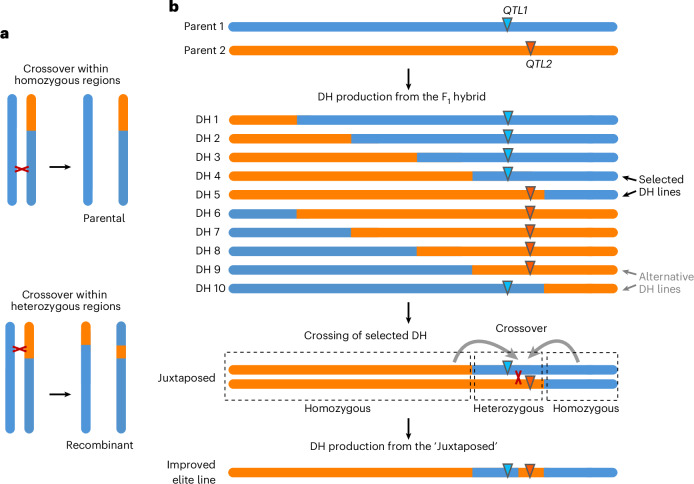
The impact of the juxtaposition effect on generating genetic variability and its application in crop breeding. **a**, Stimulating crossovers in heterozygous regions enables the creation of more allelic combinations in populations with high levels of homozygosity (that is, with high inbreeding coefficient). When large chromosomal segments are homozygous, crossovers within these regions produce gametes that retain the parental allele configuration (top panel). By contrast, crossovers in heterozygous regions always result in recombinant gametes, generating novel allelic combinations (bottom panel). **b**, Potential application of the juxtaposition effect in commercial crop breeding. The diagram illustrates a scenario in which a breeder aims to transfer valuable variation (*QTL1*) from one parental line into another. After crossing, doubled haploids are generated, each representing a unique recombinant product of the two parents. Due to the short genetic distance between *QTL1* (from parent 1) and *QTL2* (from parent 2), the likelihood of obtaining a recombinant is very low. Selection of doubled haploid (DH) lines based on polymorphism patterns allows identification of ideal partners for subsequent crosses in the breeding program. The resulting progeny show a Juxtaposed configuration in the region encompassing *QTL1* and *QTL2*, substantially increasing the chances of obtaining a recombinant. Alternative doubled haploid lines are shown in grey; these facilitate the QTL1/QTL2 combination while retaining a genetic background predominantly from parent 1. A second round of doubled haploid production facilitates the development of a new elite line enriched with *QTL1*.

The separation of dicotyledonous *Arabidopsis* and monocotyledonous maize by approximately 140 million years of evolution^57^, coupled with the conservation of key features of this process—such as its dependence on polymorphism patterns along the chromosome and independence from genetic background—suggests that the juxtaposition effect may be universal among all flowering plants. From an evolutionary perspective, this mechanism may be beneficial in populations with a high level of inbreeding, where a large portion of the genome is homozygous. Given the typically low number of crossovers in most eukaryotes^58^, stimulating crossovers in heterozygous regions enhances the generation of novel allelic combinations within the population. In the extreme scenario where only a single chromosomal region is heterozygous, any crossover within that region will consistently produce new allele combinations, whereas crossovers within homozygous regions will invariably recreate parental haplotypes (Fig. 6a). Importantly, in *Arabidopsis*, the chromosomal distribution of crossovers has been shown to be highly similar between hybrids and inbred lines^19,59^, indicating that a heterogeneous pattern of polymorphism along the chromosome is necessary to elicit this effect.

Meiotic crossover is crucial for plant breeding, enabling the development of novel haplotypes that combine beneficial alleles during the pre-breeding of elite crop varieties^7,60^. While practical crossover frequency can be globally increased through genetic modifications^60,61^, the benefits of this approach for breeding remain debated^62,63^. Moreover, such strategies typically require mutations in DNA repair genes, which often lead to chromosomal segregation defects, particularly in cereals^64,65^. An alternative strategy, widely considered optimal for breeding, involves directing crossover events to specific chromosomal regions^7,8^. However, no such method has yet been developed for plants. In this study, we demonstrate that a similar outcome can be achieved without genetic modifications by selecting parental lines that show specific heterozygosity patterns around target genomic regions (Fig. 6b). Because the effect manifests only when a large proportion of the chromosome is homozygous, this strategy is particularly complementary to doubled-haploid technology, which is widely used for the rapid production of inbred lines. We see the juxtaposition effect as especially useful when linkage prevents recombination between two closely linked quantitative trait loci (QTLs) in a single genotype (Fig. 6b)—selecting optimal doubled haploid lines for crossing can multiply the probability of obtaining the desired genotype. As the effect likely relies on crossover redistribution from adjacent homozygous regions (see conceptual models on Figs. 2h,i and 5g), it could potentially be also exploited to locally suppress recombination and preserve linkage between beneficial QTLs. An open question remains whether the juxtaposition effect can also be harnessed to unlock genetic variation in recombination-suppressed pericentromeric regions.

In maize, this approach increased local crossover frequency by up to an unprecedented threefold. We did not observe differences in Rf stimulation between two maize backgrounds differing with the polymorphism density, suggesting that the strength of the effect is not affected by the polymorphism density. Hence, we propose that this phenomenon should be integrated into the design of breeding strategies. In the era of high-throughput sequencing, where thousands of fully characterized lines are readily available, this discovery offers a powerful tool for breeders to accelerate the transfer of beneficial traits and enhance selection efficiency (Fig. 6b).

## Methods

### Generation of lines and assessment of Rf in *Arabidopsis*

The R^2^-BT line was achieved by crossing a recombinant from Col-BT × L*er*-0 cross, which carried a dsRed marker expressed in seeds, with another recombinant carrying an eGFP reporter, both selected based on previously identified crossover breakpoints^18^. Double-reporter individuals homozygous for one transgene and hemizygous for the other (GR/-R or GR/G-) were selected using an epifluorescence stereomicroscope (Lumar ver. 12, Zeiss) and backcrossed to the Col background, resulting in the R^2^-BT line. The scheme illustrating the generation of the R^2^-BT line is presented in Supplementary Fig. 1.

Recombination within the R^2^-BT lines was assessed using a seed-based system as outlined in the published protocol^16^^,18^. Homozygous fluorescently tagged lines were crossed with the non-colour accessions, and images of F_2_ seeds derived from entire plants were captured using the epifluorescence stereomicroscope. Each image set included brightfield as well as ultraviolet images through red and green fluorescence filters. Seed counting was performed using the CellProfiler v2.1.1., following the established protocol^66^. Due to low recombination rates within the intervals, single-colour recombinant seeds were identified manually. To improve the accuracy of crossover measurements within these short intervals, all seeds from each plant were analysed, yielding an average sample size of ~3,900–10,000 seeds per biological replicate. The Rf in centimorgans (cM) was calculated using the following formula:

$${\rm{Rf}}=100\left(1-\sqrt{1-\frac{2\left(g+r\right)}{n}}\right),$$

where *g* represents the number of green-only seeds, *r* the number of red-only seeds, and *n* the total number of seeds per plant. Raw seed scoring data for all measurements are presented in Supplementary Table 2.

### Generation of phenotypic reporter lines for Rf assessment in maize

To develop maize NILs, the inbred line 2-9m, characterized by nine linked mutant alleles for phenotypic traits (Supplementary Table 4), was used as the male parent in crosses with two inbred lines, Ku123 and MK01. The multimarker line 2-9m was developed in the early 1980s in Moldova from a cross between two previously established lines carrying markers on chromosome 2 (*ws3*,*lg1*,*gl2*), and chromosomes 9 and 10 (*c1*,*sh1*,*wx1*,*R1*). These parental lines carried dominant alleles at the loci of interest, except for the recessive *r1* allele^31^. After two rounds of self-pollination with phenotypic selection, four to six backcrosses were performed using either MK01 or Ku123 as the recurrent parent (Fig. 1i and Supplementary Table 12).

At each generation, phenotypic selection was based on grain or seedling traits, and only plants retaining the target marker pair in the genome (for example, *c1–sh1* in Fig. 1i or *lg1–gl2* in Extended Data Fig. 2) were advanced to the next backcrossing cycle. NILs were systematically labelled: ‘M’ or ‘Ku’ indicated the MK01 or Ku123 genetic background, respectively, and transferred mutant alleles were listed in parentheses.

This strategy generated 12 NILs, including the following: M(*c1*,*sh1*,*R1*), M(*sh1*,*wx1*), M(*c1*,*sh1*,*wx1*,*R1*), M(*lg1*,*gl2*), M(*ws3*,*lg1*), M(*ws3*,*gl2*), M(*ws3*,*lg1*,*gl2*), M(*R1*), Ku(*c1*,*sh1*,*R1*), Ku(*sh1*,*wx1*), Ku(*lg1*,*gl2*), Ku(*R1*). In addition, the recessive *c1* and dominant *R1* alleles were incorporated to enable anthocyanin pigmentation in kernels, simplifying the tracking of segregation at the *c1* locus. Consequently, M(*R1*) and Ku(*R1*) lines were developed for hybridization with NILs instead of MK01 or Ku123 when analyses specifically targeting the *c1* locus were required.

### Assessment of Rf in maize NILs

The Rf in F_1_ hybrid plants of NILs, carrying intervals *c1–sh1*, *sh1–wx1* and *lg1–gl2*, was assessed using backcross families derived from crossing these NIL F_1_ hybrids with their respective NIL parental lines. These backcross families were generated with the NIL F_1_ hybrids serving as either the female or male parent. The presence of the marker (mutant allele) was determined either by visual inspection of kernels on the ear—*c1* (colourless aleurone layer), *sh1* (shrunken endosperm) and *wx1* (waxy endosperm)—or by observing phenotypes in seedlings grown from the plant’s seeds—*ws3* (white leaf sheath and husk), *lg1* (liguleless leaves) and *gl2* (glossy leaves that retain water droplets).

The Rf between two markers in the backcross families was calculated using the following formula:

$${\rm{Rf}}=\frac{b+c}{n},$$

where *b* and *c* represent the number of recombinant progenies, and *n* represents the total progeny number in the family (*n* = *a* + *b* + *c* + *d*). *a* and *d* are the numbers of non-recombinant progenies. The error associated with the Rf was calculated using the following formula:

$$e=\frac{\sqrt{\left(b+c\right)\left(a+d\right)}}{n\sqrt{n}}.$$

For the NILs M(*ws3*,*lg1*), M(*lg1*,*gl2*) and M(*ws3*,*gl2*), Rf was estimated using F_2_ populations and maximum likelihood estimation, which reduces estimation error when certain genotypic classes are missing or very small^67,68^. The Rf was calculated using the following formula:

$${\rm{Rf}}=1-\sqrt{\theta },$$

where $\theta$ is the product of non-recombination probabilities in male and female meiosis, determined from the following quadratic equation:

$$n{\theta }^{2}-\left(a-2b-2c-d\right)\theta -2d=0,$$

which can be solved as follows:

$$\theta =\frac{a-2b-2c-d\pm \sqrt{{\left(a-2b-2c-d\right)}^{2}+8{nd}}}{2n}.$$

This estimate corresponds to the geometric mean of recombination in male and female meiosis, where

$$\theta =\left(1-{\rm{Rf}}_{\rm{male}}\right)\left(1-{\rm{Rf}}_{\rm{female}}\right).$$

The error in this estimate was calculated as follows:

$$e=\sqrt{\frac{\left(1-\theta \right)\left(2+\theta \right)}{2n\left(1+2\theta \right)}}.$$

The average Rf for each genotype was obtained by averaging the Rf values of families (replicates) containing more than 50 individuals.

For the three-point crosses, the average Rf between male and female was calculated and plotted alongside the F_2_ data for the genotypes M(*R1*) × M(*c1*,*sh1*,*wx1*,*R1*), Ku(*R1*) × M(*c1*,*sh1*,*wx1*,*R1*), M × M(*ws3*,*lg1*,*gl2*) and Ku(*R1*) × M(*ws3*,*lg1*,*gl2*) (Fig. 3f,g). When more data were available for one sex, a random subset was selected from the larger group to match the sample size of the other, ensuring a balanced calculation of the average Rf.

### High-molecular-weight DNA extraction and long-read sequencing

Four 10-day-old Ku123 seedlings, grown in the dark at 21 °C and 70% humidity, were collected and ground in liquid nitrogen. High-molecular-weight DNA was extracted using the Nucleobond HMW DNA kit (Macherey-Nagel, 740160.20) according to the manufacturer’s protocol. DNA quality was checked on a 0.6% agarose gel, and samples were outsourced for library preparation and sequencing using a PacBio HiFi Revio.

### Ku123 genome assembly

The draft genome assembly of the maize line Ku123 was generated using hifiasm v0.19.8 with default parameters^69^ from PacBio HiFi long reads. Collinearity of the resulting contigs was assessed against the reference genome assemblies B73 v5 (ref. ^33^) and Mo17 (ref. ^34^) using D-Genies v1.5.0 (ref. ^70^), with ‘Minimap2 v2.28’ and ‘many repeats’ settings. N90 contigs were oriented and assembled into pseudomolecules with 100 ‘N’ gaps based on their collinearity with B73 v5 and Mo17 v2 genomes. The final de novo genome assembly, including pseudomolecules, was subsequently compared to the B73 v5 and Mo17 v2 reference genomes using SyRI v1.7.0 (ref. ^71^), after masking the Ku123 genome for repetitive sequences, using bedtools v2.30.0 with the parameters ‘maskfasta -mc N’^72^.

### Assessment of DNA polymorphism by Illumina sequencing

For each genotype, a single plant was grown for 10 days in a controlled environment chamber set to 21 °C, under long-day conditions (16 h light/8 h dark), with 70% humidity and a light intensity of 150 μmol. In the case of certain maize NILs, the subsequent generation was used, as the generation originally assessed for Rf was not available. DNA extraction was performed following the protocol outlined by ref. ^73^, and DNA quality was verified on a 1% agarose gel. Library preparation was conducted as described by ref. ^19^. Briefly, tagmentation was carried out by mixing 1 µl of 5 ng µl^−1^ DNA with 1 µl of tagmentation buffer (40 mM Tris–HCl, pH 7.5; 40 mM MgCl_2_), 0.5 µl of DMF (Sigma, 68-12-2), 2.35 µl of nuclease-free water (Thermo Fisher, R0581) and 0.05 µl of pre-loaded, in-house-produced Tn5 transposase. The loading of Tn5 with annealed linker oligonucleotides was previously described. The tagmentation reaction was incubated at 55 °C for 2 min and terminated by adding 1 µl of 0.1% SDS, followed by a 10 min incubation at 65 °C.

Amplification of the tagmented DNA was performed using the KAPA2G Robust PCR kit (Sigma, 2GRKB) with custom P5 and P7 indexing primers, ensuring each sample was amplified with a unique set of primers, as described by ref. ^73^. Successfully amplified libraries were pooled and size-selected using 2% agarose gel electrophoresis. DNA fragments between 400 and 700 bp were excised and extracted using the Gel Extraction Kit (ZymoResearch, D4008). Paired-end sequencing was then carried out on a NovaSeq X Plus (Illumina). Sequencing quality was assessed using FastQC v0.12.1 with default parameters.

Reads were mapped to the Ku123v1 assembly using Bowtie2 v2.2.3 (ref. ^74^) with the parameter ‘—trim5 20’ to remove 20 low-quality nucleotides from the 5′ ends of each read. Duplicate reads were removed from the resulting BAM file using Samtools v1.3.1 ‘rmdup’. SNP calling was performed with Samtools v1.3.1 ‘mpileup -u -f’ and Bcftools v1.13 ‘-v -c’^75^, and SNP densities were calculated using VCFtools v0.1.17 (ref. ^76^). All plotting and data visualizations were done using R software v4.1.2 (ref. ^77^).

### Centromere location determination

The positions of centromere-specific repeats, including CentC, CRM1, CRM2, CRM3 and CRM4, were identified using BLASTn v2.9.0 (ref. ^78^) based on the high-quality maize transposable element library, maizeTE02052020 from the Maize TE Consortium. The centromere borders were determined visually by analysing the density of these specific repeats per megabase along the chromosomes.

### Annotation of repetitive elements and genes

Transposable elements and repetitive elements annotation was conducted using the EDTA v2.2.1 software^79^ and the curated library from the Maize TE Consortium (maizeTE02052020), with the parameter ‘–species Maize’ and classified according to ref. ^80^. Any ‘unknown’ entries in the transposable element categories were excluded from further analysis. Gene prediction was performed ab initio using Augustus v3.5.0 online software^81^ with the pre-existing *Zea mays* parameters. Any gene predictions overlapping with transposable elements were removed using bedtools v2.30.0 ‘intersect -v’^72^.

### Annotation of nuclear mitochondrial DNA (NUMTs) and nuclear plastid DNA (NUPTs) integration

Organellar DNA sequence of chloroplasts and mitochondria from B73 v5 (ref. ^71^) were aligned on the Ku123 v1 genome assembly using minimap2 v2.28.-r1209 (ref. ^82^) with default parameters. For mitochondria and chloroplasts, alignments of at least 5 kb and 95% identity and alignments of at least 3 kb and 95% identity were kept, respectively, as described in ref. ^83^. Contiguous alignment results with a distance of less than 100 kb were merged to generate regions of integrated organellar genome (Supplementary Table 11).

### Genomic position of the phenotypic markers

Gene sequences corresponding to the phenotypic markers were obtained from MaizeGDB^32^, and their positions on the Ku123 v1 assembly were identified using BLASTn v2.9.0 (ref. ^78^). As no gene model was available for the *ws3* phenotypic marker, the two genetically closest genes, *mlo9* (Zm00001eb065900) and *pco061701* (Zm00001eb065940), located upstream and downstream of *ws3* on the MaizeGDB composite genetic map, were used. The midpoint between these two genes was estimated as the approximate physical position of *ws3*. All positions are available in Supplementary Table 4.

### Statistical analysis

Rf differences between genotypes in *Arabidopsis* and maize were analysed by first assessing normality and homoscedasticity using the Shapiro–Wilk test and *F*-test, respectively. For datasets meeting these assumptions, a two-tailed Welch’s *t*-test was used for mean comparison. If normality was not satisfied, the non-parametric Mann–Whitney *U*-test was applied. For multiple comparisons of Rf in *Arabidopsis* (Fig. 1d) and maize (Fig. 3f,g), Welch’s analysis of variance (ANOVA) followed by the Games–Howell post hoc test was used, as the assumption of homoscedasticity was not met for all groups. All statistical analyses were conducted with a significance threshold of *α* = 0.05, and detailed results are provided in Supplementary Tables 3, 15 and 17.

### Reporting summary

Further information on research design is available in the Nature Portfolio Reporting Summary linked to this article.

## Supplementary information

Supplementary Information Supplementary Figs. 1 and 2.

Reporting Summary

Supplementary Tables Supplementary Tables 1–18.

## Figures and Tables

**Extended Data Fig. 10:**
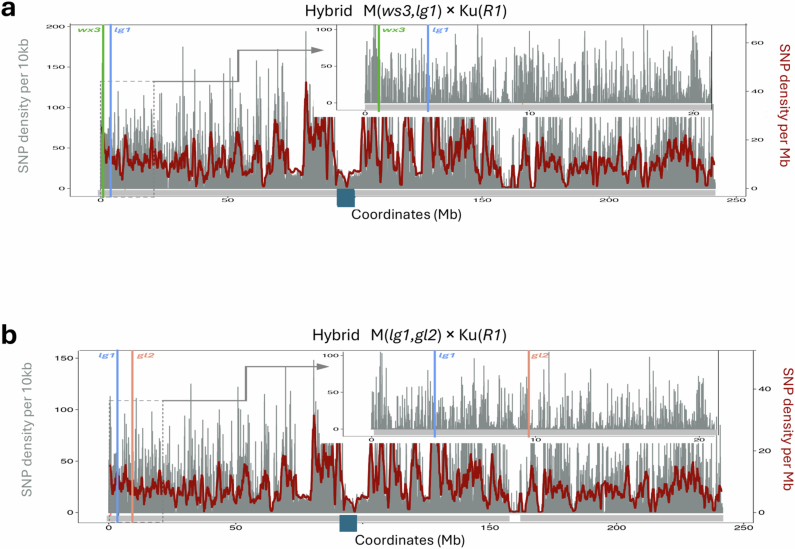
Short homozygous blocks interrupting the heterozygous region suppress local crossover stimulation. **a**, Interhomolog SNP distribution in the M(*ws3,lg1*) × Ku(*R1*) cross along chromosome 2. Gray vertical bars represent SNP density per 10 kb, with the burgundy trend line showing SNP density per 1 Mb. Gray horizontal bars beneath the graph indicate estimated heterozygous regions based on SNPs. Colored lines mark the positions of the *ws3-lg1* interval used to measure Rf. The navy blue square on the x-axis shows the centromere location. The inset offers a close-up of the interval, with the SNP density plots below partially trimmed. **b**, As in (**a**), but for the M(*lg1*,*gl2*) × Ku(*R1*) cross.

## Data Availability

All data generated for this study are included in the published version of the article or its Supplementary Information. The Ku123 genome assembly and the genotyping-by-sequencing data have been deposited in the National Center for Biotechnology Information (NCBI) Sequence Read Archive under the BioProject accession code PRJNA1186655. Raw genotyping-by-sequencing data for the wild-type Col × L*er* F_2_ population were downloaded from ArrayExpress E-MTAB-816541. The Col-0 TAIR10 reference genome is downloaded from the TAIR database. The sequence polymorphism data for the Col/L*er* cross used in this study were downloaded from 1001 Genomes. Rf raw data generated in this study are provided in Supplementary Tables 2, 5 and 14. The genome assembly of maize B73 v5 was downloaded from MaizeGDB. The maize Mo17v2 genome assembly was downloaded from the NCBI BioProject PRJNA751841. The genome assembly of maize A188 was downloaded from MaizeGDB. The genome assembly of the maize variety W22 was downloaded from the NCBI accession number GCA_001644905.2. The source data file is available via Zenodo at 10.5281/zenodo.15862594 (ref. ^84^). All unique materials generated in this study are available from the authors.
