# Supplementary material for: Enhancing local meiotic crossovers in Arabidopsis and maize through juxtaposition of heterozygous and homozygous regions
Source: Nat Plants. 2025 Sep 2;11(9):1769–84. doi: 10.1038/s41477-025-02085-8 (PMC12449268; doi:10.1038/s41477-025-02085-8)
Supplement: Supplementary file 1 — Supplementary Figs. 1 and 2. [file 41477_2025_2085_MOESM1_ESM.pdf]

nature plants

Supplementary information https://doi.org/10.1038/s41477-025-02085-8

Enhancing local meiotic crossovers in *Arabidopsis* and maize through juxtaposition of heterozygous and homozygous regions

In the format provided by the authors and unedited

Table of Content

1. Supplementary Figure 1
2. Supplementary Figure 2

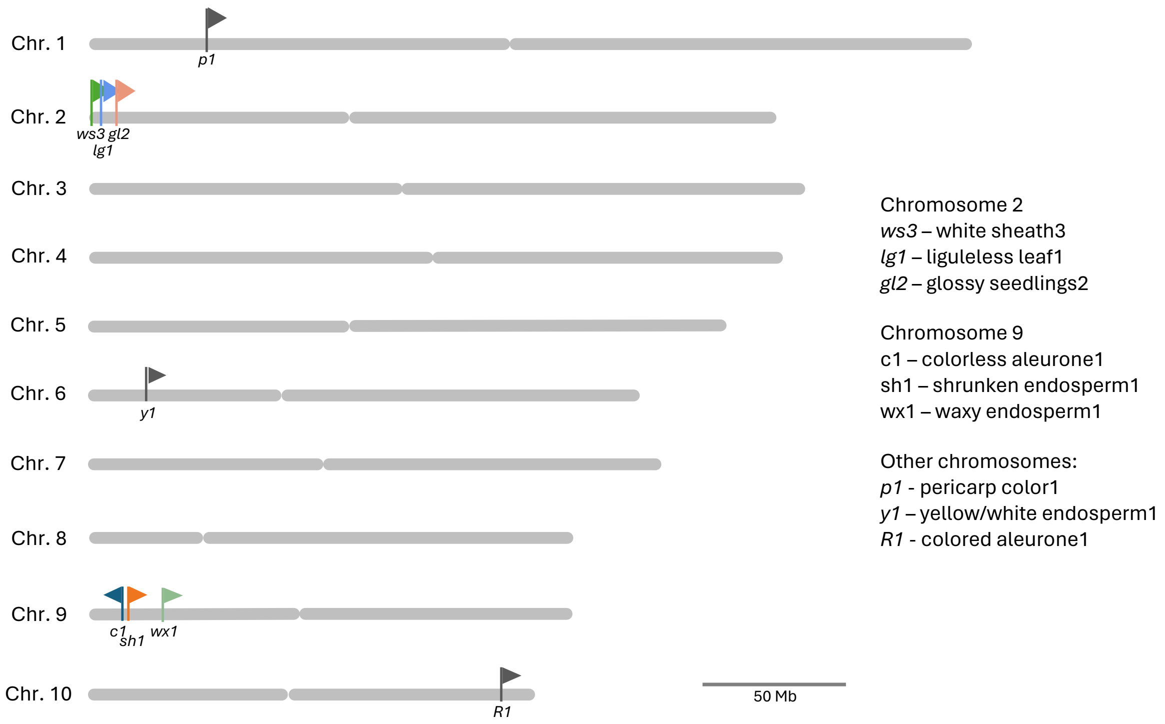

Supplementary Figure 1. Physical location of the phenotypic mutant markers in the 2-9m genome. Marker pairs *ws3-lg1*, *lg1-gl2*, *ws3-gl2* on chromosome 2, along with *c1-sh1*, *sh1-wx1* on chromosome 9, were used to define intervals for recombination frequency (Rf) measurements (indicated by color flags). Markers *p1* and *y1* were not used in the study (indicated by gray flags). *R1* marker on chromosome 10 was used to reveal the phenotype of the *c1* marker (See Online Methods). The locations are given on a megabase scale as mapped to the Ku123 genome assembly (this study).

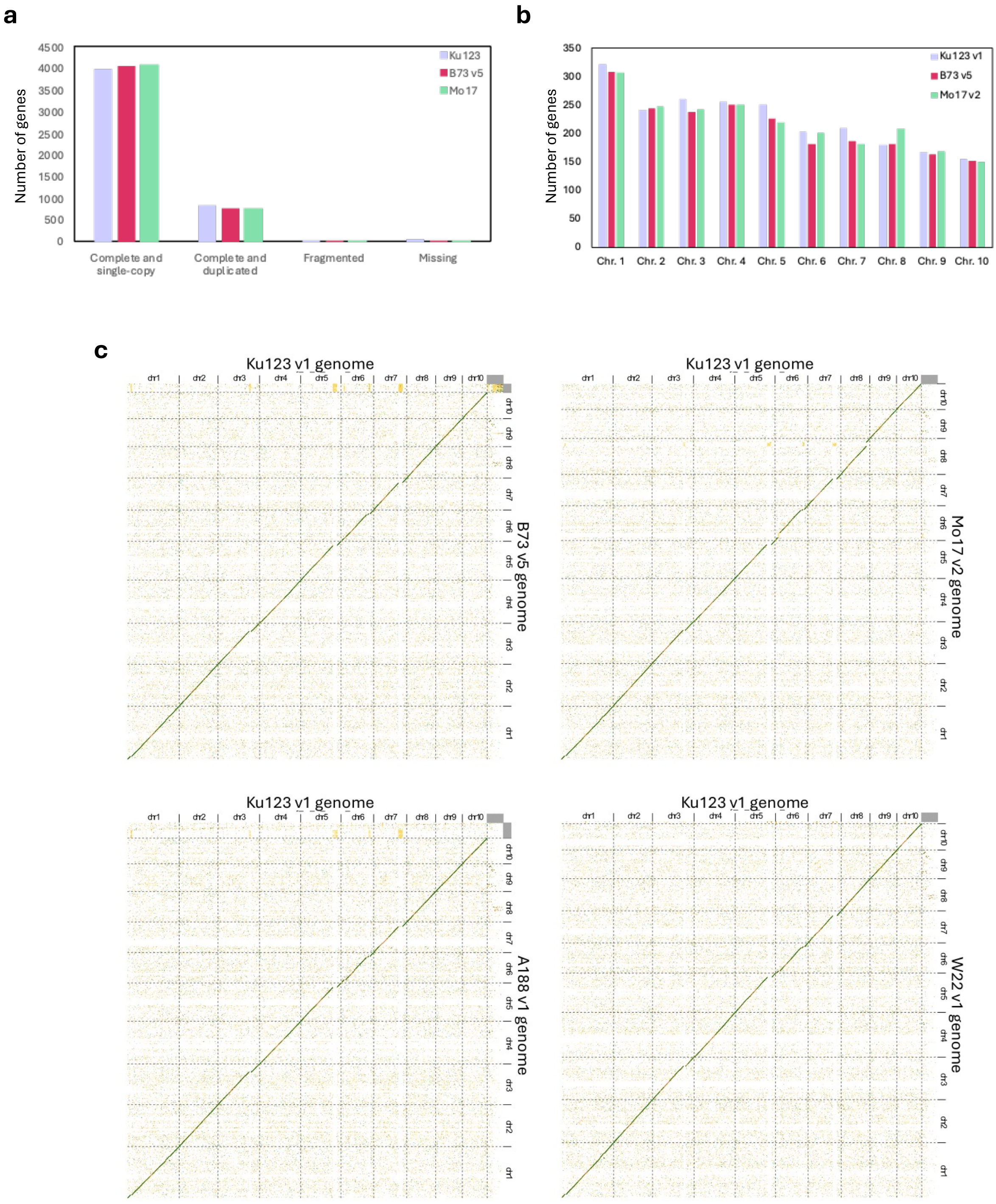

Supplementary Figure 2. Characterization of the Ku123 genome in terms of its completeness compared to the reference genomes B73 v5 and Mo17. a, BUSCO analysis showing the identified conserved genes in the Ku123 genome, with an emphasis on their completeness. **b**, Comparison of chromosome length between the two maize assemblies. **c**, Collinearity of the Ku123 *de novo* genome assembly with the B73 v5, Mo17 v2, A188 v1 and W22 v1 maize genomes, presented as dot plots.
