# Supplementary material for: Enhancing local meiotic crossovers in Arabidopsis and maize through juxtaposition of heterozygous and homozygous regions
Source: Nat Plants. 2025 Sep 2;11(9):1769–84. doi: 10.1038/s41477-025-02085-8 (PMC12449268; doi:10.1038/s41477-025-02085-8)
Supplement: Supplementary file 2 — Reporting Summary [file 41477_2025_2085_MOESM2_ESM.pdf]

nature portfolio

Corresponding author(s): Piotr A. Ziolkowski
Mikhail E. Mikhailov

Last updated by author(s): Jul 11, 2025

Reporting Summary

Nature Portfolio wishes to improve the reproducibility of the work that we publish. This form provides structure for consistency and transparency in reporting. For further information on Nature Portfolio policies, see our Editorial Policies and the Editorial Policy Checklist.

Statistics

For all statistical analyses, confirm that the following items are present in the figure legend, table legend, main text, or Methods section.

n/a	Confirmed	
☐	☒	The exact sample size (*n*) for each experimental group/condition, given as a discrete number and unit of measurement
☐	☒	A statement on whether measurements were taken from distinct samples or whether the same sample was measured repeatedly
☐	☒	The statistical test(s) used AND whether they are one- or two-sided<br>*Only common tests should be described solely by name; describe more complex techniques in the Methods section.*
☐	☒	A description of all covariates tested
☐	☒	A description of any assumptions or corrections, such as tests of normality and adjustment for multiple comparisons
☐	☒	A full description of the statistical parameters including central tendency (e.g. means) or other basic estimates (e.g. regression coefficient) AND variation (e.g. standard deviation) or associated estimates of uncertainty (e.g. confidence intervals)
☐	☒	For null hypothesis testing, the test statistic (e.g. *F*, *t*, *r*) with confidence intervals, effect sizes, degrees of freedom and *P* value noted<br>*Give P values as exact values whenever suitable.*
☒	☐	For Bayesian analysis, information on the choice of priors and Markov chain Monte Carlo settings
☒	☐	For hierarchical and complex designs, identification of the appropriate level for tests and full reporting of outcomes
☒	☐	Estimates of effect sizes (e.g. Cohen's *d*, Pearson's *r*), indicating how they were calculated

Our web collection on statistics for biologists contains articles on many of the points above.

Software and code

Policy information about availability of computer code

Data collection	No software was used to collect the data. The software used to process the pictures of seeds for crossover frequency measurements in Arabidopsis was CellProfiler (version 4.07), which is freely available (https://cellprofiler.org).
Data analysis	The following softwares were used to analyse the data: hifiasm v0.19.8, SyRI v1.7.09, bedtools v2.30.0 bowtie2 v2.5.4, samtools v1.3.1, VCFtools v0.1.1715, D-Genies v1, R software v4.1.216, BLASTn v2.9.0, EDTA v2.2.1, minimap2 v2.28. The detailed use of these softwares are presented in the Online Methods section of the manuscript.

For manuscripts utilizing custom algorithms or software that are central to the research but not yet described in published literature, software must be made available to editors and reviewers. We strongly encourage code deposition in a community repository (e.g. GitHub). See the Nature Portfolio guidelines for submitting code & software for further information.

Data

Policy information about availability of data

All manuscripts must include a data availability statement. This statement should provide the following information, where applicable:

- Accession codes, unique identifiers, or web links for publicly available datasets
- A description of any restrictions on data availability
- For clinical datasets or third party data, please ensure that the statement adheres to our policy

All data generated for this study are included in the published version of the article, or its Supplementary Material. The Ku123 genome assembly and the GBS

sequence data generated in this study have been deposited in the NCBI Sequence Read Archive (SRA) under the BioProject accession codes PRJNA1186655. Raw GBS data for the wild-type Col×Ler F2 population were downloaded from ArrayExpress E-MTAB-816541. The Col-0 TAIR10 reference genome is downloaded from the TAIR database. The sequence polymorphism data for the Col/Ler cross used in this study was downloaded from 1001 Genomes. Rf raw data generated in this study are provided in the Supplementary Tables 2, 5 and 14. The genome assembly of maize B73 v5 was downloaded from MaizeGDB. The maize Mo17v2 genome assembly was downloaded from the NCBI BioProject PRJNA751841. The genome assembly of maize A188 was downloaded from MaizeGDB. The genome assembly of the maize variety W22 was downloaded from the NCBI accession number GCA_001644905.2. All unique materials generated in this study are available from the authors.

Research involving human participants, their data, or biological material

Policy information about studies with human participants or human data. See also policy information about sex, gender (identity/presentation), and sexual orientation and race, ethnicity and racism.

Reporting on sex and gender	N/A
Reporting on race, ethnicity, or other socially relevant groupings	N/A
Population characteristics	N/A
Recruitment	N/A
Ethics oversight	N/A

Note that full information on the approval of the study protocol must also be provided in the manuscript.

Field-specific reporting

Please select the one below that is the best fit for your research. If you are not sure, read the appropriate sections before making your selection.

☒ Life sciences ☐ Behavioural & social sciences ☐ Ecological, evolutionary & environmental sciences

For a reference copy of the document with all sections, see nature.com/documents/nr-reporting-summary-flat.pdf

Life sciences study design

All studies must disclose on these points even when the disclosure is negative.

Sample size	Sample sizes were chosen based on our previous experience — for Arabidopsis thaliana (Ziolkowski et al., 2015; Kbiri et al., 2022; Dluzewska et al., 2023; Szymanska-Lejman et al., 2023) and for maize (Mikhailov & Chernov, 2004) — and our understanding of the sample size required to reliably detect the expected effect size.
Data exclusions	For Arabidopsis thaliana recombination frequency calculations performed with seed-based system, samples with color/non-color seeds ratio that did not fall within 2.7-3.3 value (based on Mendelian segregation of fluorescent markers), were excluded from analysis.<br>For maize, replicates presenting less than 50 seeds were discarded following our pre-established exclusion threshold, as samples presenting less seeds that might not be representative and underestimate the recombination frequency.
Replication	For Arabidopsis, recombination frequency for each genotype was calculated using data from at least nine individuals, each contributing between 1,477 and 8,450 seeds. All replication attempts were successful.<br>In maize, recombination frequency for each genotype was calculated using data from at least three individuals, with each individual contributing between 56 and 958 seeds. All replication attempts were successful.
Randomization	Randomization of sample allocation into groups was not applicable, as the research question required precise contrasts between 'Hybrid' and 'Juxtaposed' heterozygosity. Group assignments were therefore based on known parental genotypes and targeted interval polymorphisms.
Blinding	To minimize observer bias, crossover measurements were conducted using a single-blind approach, with genotypes concealed from the observer and revealed only upon completion of the measurements.

Reporting for specific materials, systems and methods

We require information from authors about some types of materials, experimental systems and methods used in many studies. Here, indicate whether each material, system or method listed is relevant to your study. If you are not sure if a list item applies to your research, read the appropriate section before selecting a response.

Materials & experimental systems

n/a	Involved in the study
☒	☐ Antibodies
☒	☐ Eukaryotic cell lines
☒	☐ Palaeontology and archaeology
☒	☐ Animals and other organisms
☒	☐ Clinical data
☒	☐ Dual use research of concern
☐	☒ Plants

Methods

n/a	Involved in the study
☒	☐ ChIP-seq
☒	☐ Flow cytometry
☒	☐ MRI-based neuroimaging

Plants

Seed stocks	The seeds of the Arabidopsis ecotypes Col-0 and Ler-0 were obtained from the European Arabidopsis Stock Centre (uNASC), under accession numbers N1093 and NW20, respectively. The Arabidopsis Col-BT line was described in Szymanska-Lejman et al. (2023). The maize parental lines Ku123, Mk01, and 2-9m were obtained from the Institute of Genetics, Physiology and Plant Protection, Moldova State University, Chisinau, Moldova.
Novel plant genotypes	Arabidopsis:<br>The Arabidopsis R2-BT line was generated by crossing a recombinant from a Col-BT × Ler-0 cross (carrying a dsRed marker) with a recombinant carrying an eGFP reporter. Double-reporter individuals that were homozygous for one transgene and hemizygous for the other (GR/-R or GR/G-) were selected and backcrossed to the Col-0 background.<br>Maize:<br>To develop maize near-isogenic lines (NILs), the inbred line 2-9m was used as the male parent in crosses with two other inbred lines, Ku123 and MK01. After two generations of self-pollination with phenotypic selection, 4 to 6 backcrosses were carried out using either MK01 or Ku123 as the recurrent parent, with phenotypic selection applied at each generation. NILs were systematically labeled: “M” or “Ku” indicated the MK01 or Ku123 genetic background, respectively, and the transferred mutant alleles were listed in parentheses. This approach produced 12 NILs, including: M(c1, sh1, R1), M(sh1, wx1), M(c1, sh1, wx1, R1), M(lg1, gl2), M(ws3, lg1), M(ws3, gl2), M(ws3, lg1, gl2), M(R1),Ku(c1, sh1, R1), Ku(sh1, wx1), Ku(lg1, gl2), and Ku(R1).
Authentication	Each genotype generated in this study (both for Arabidopsis and maize) were characterized using phenotypic markers and Illumina short reads sequencing.
